# Contributions of deep learning to automated numerical modelling of the interaction of electric fields and cartilage tissue based on 3D images

**DOI:** 10.3389/fbioe.2023.1225495

**Published:** 2023-08-29

**Authors:** Vien Lam Che, Julius Zimmermann, Yilu Zhou, X. Lucas Lu, Ursula van Rienen

**Affiliations:** ^1^ Institute of General Electrical Engineering, University of Rostock, Rostock, Germany; ^2^ Department of Mechanical Engineering, University of Delaware, Delaware, DE, United States; ^3^ Department Life, Light and Matter, University of Rostock, Rostock, Germany; ^4^ Department of Ageing of Individuals and Society, Interdisciplinary Faculty, University of Rostock, Rostock, Germany

**Keywords:** machine learning, deep learning, image segmentation, bioimpedance, numerical models, electrical stimulation, computational electromagnetics

## Abstract

Electric fields find use in tissue engineering but also in sensor applications besides the broad classical application range. Accurate numerical models of electrical stimulation devices can pave the way for effective therapies in cartilage regeneration. To this end, the dielectric properties of the electrically stimulated tissue have to be known. However, knowledge of the dielectric properties is scarce. Electric field-based methods such as impedance spectroscopy enable determining the dielectric properties of tissue samples. To develop a detailed understanding of the interaction of the employed electric fields and the tissue, fine-grained numerical models based on tissue-specific 3D geometries are considered. A crucial ingredient in this approach is the automated generation of numerical models from biomedical images. In this work, we explore classical and artificial intelligence methods for volumetric image segmentation to generate model geometries. We find that deep learning, in particular the *StarDist* algorithm, permits fast and automatic model geometry and discretisation generation once a sufficient amount of training data is available. Our results suggest that already a small number of 3D images (23 images) is sufficient to achieve 80% accuracy on the test data. The proposed method enables the creation of high-quality meshes without the need for computer-aided design geometry post-processing. Particularly, the computational time for the geometrical model creation was reduced by half. Uncertainty quantification as well as a direct comparison between the deep learning and the classical approach reveal that the numerical results mainly depend on the cell volume. This result motivates further research into impedance sensors for tissue characterisation. The presented approach can significantly improve the accuracy and computational speed of image-based models of electrical stimulation for tissue engineering applications.

## 1 Introduction

Articular cartilage, also known as hyaline cartilage, is a heterogeneous and hierarchical arrangement of an avascular tissue that covers opposing skeletal ends in diarthrodial joints (Cohen et al., 1998; Fox et al., 2009). It is composed of chondrocytes and their surrounding pericellular matrix (PCM) enclosed in an extracellular matrix (ECM) (Jahr et al., 2015). The chondrocytes are accountable for synthesising and maintaining the ECM, which is made of collagen networks, charged proteoglycan gels, and other proteins. The chondrocytes also release substances that contribute to the flexibility and strength of cartilage. Three distinct zones of articular cartilage can be distinguished based on different matrix compositions and cellular characteristics, namely, the superficial (top), transitional (middle), and radial (deep) zones (Hunziker et al., 2002; Martel-Pelletier et al., 2008; Fox et al., 2009).

Upon progression of osteoarthritis, which is a severely detrimental condition caused by the degeneration of cartilage, the composition and structure of the tissue are altered (Maldonado and Nam, 2013; Chen et al., 2017). However, human cartilage is nearly incapable of self-healing, meaning injuries tend to worsen with time (Keeney et al., 2011). As a result, osteoarthritis has been a significant contributor to the global burden of disease (Nelson, 2018). Electrical stimuli are being studied as a potential means of enhancing proliferation, differentiation, and cell activity in cartilaginous tissue regeneration (Brighton et al., 2008; Vaca-González et al., 2017; Krueger et al., 2021). Despite experimental progress, a deep understanding of the effect of electrical stimulation on cartilage is still lacking. The lack of understanding hampers patient-specific tissue engineering and regenerative medicine approaches for the treatment of osteoarthritis. In this regard, fine-grained numerical simulations provide insight into the interaction between cells and electric fields. The induced cellular transmembrane potential as a measure of the stimulation dosage can be estimated (Zimmermann et al., 2022a). Moreover, the ambiguity of the dielectric properties of biological tissue in general and cartilage in particular can be resolved (Zimmermann and van Rienen, 2021; Zimmermann et al., 2022b). In turn, the fine-grained numerical models including the cellular scale can be used to calibrate coarser volume conductor models at the tissue scale.

Fine-grained models have to capture the cellular organisation, cell distributions and their anatomical representation in the tissue. Previously, confocal fluorescence microscopy images have been used to create tissue-specific geometries and meshes (Bennetts et al., 2014). In this workflow, the geometry and mesh generation has been automated and made available as open-source software. Nevertheless, the approach described by Bennetts et al. (2014) requires segmented images and cells that can be described by ellipsoids. A manual or semi-automated image segmentation becomes prohibitively expensive with a growing sample size. Furthermore, the description of cells by ellipsoids is limited and might not always be justified. Thus, we explore how the recent developments of artificial intelligence for image segmentation can be exploited to automate the modelling approach. The goal is to generate tissue-specific models starting from the original fluorescence microscopy images with minimal user involvement.

The segmentation of 3D cellular images is highly challenging (Weigert et al., 2020; Hollandi et al., 2022; Xu et al., 2022). Thanks to machine learning and artificial intelligence, numerous algorithms have emerged, offering automated cell image segmentation solutions. Conventional machine learning algorithms, such as random forest, support vector machine and decision tree, have been made available in well-established open-source platforms for bioimage analysis problems (Arganda-Carreras et al., 2017; McQuin et al., 2018; Berg et al., 2019; Caicedo et al., 2019). They require manual good feature extractors such as, for example, intensity, texture, and shape to segment the images (LeCun et al., 2015). In recent years, deep learning-based techniques have illustrated considerable enhancement in outcomes for biomedical images (LeCun et al., 2015; Tokuoka et al., 2020; Weigert et al., 2020; Wang et al., 2022). Among them, convolutional neural networks (CNN) have achieved state-of-the-art performance due to their capability of automatically extracting image features. Most notably U-Net (Ronneberger et al., 2015; Çiçek et al., 2016) but also several other CNN-based models (Valen et al., 2016; He et al., 2017; Xu et al., 2018) have been proposed for cell segmentation. Deep learning-based methods can achieve remarkable results in segmenting cells when trained on a large, well-annotated dataset. Nonetheless, annotated data are scarce (i.e., images segmented manually or semi-automatically by experts), especially in 3D data. Furthermore, cell type and fluorescent stain variations can significantly impact cell images, rendering an inadequate performance of deep learning-based segmentation methods (Khan et al., 2020; Durkee et al., 2021; Minaee et al., 2021; Xu et al., 2022). As a solution to this challenge, data augmentation, transfer learning, and active learning have been developed. Transfer learning has demonstrated promising outcomes in cell segmentation by using a pre-trained network as a starting point and fine-tuning it for the specific task (Kraus et al., 2017; Khan et al., 2019; Majurski et al., 2019; Zhang et al., 2020). In active learning, high-value training samples are chosen from unlabeled data for annotation, as labelled data do not contribute equally to learning meaningful features (Lou et al., 2014; Smith and Horvath, 2014; Carse and McKenna, 2019). Still, low signal-to-noise ratios and dense packing of cells in fluorescence microscopy datasets pose a challenge to cell instance segmentation, locating individual cells and labelling them with different pixels in volumetric images. Currently, popular methods can be divided into anchor-based (Johnson, 2018; Tsai et al., 2019; Hollandi et al., 2020) and region-based methods (Kainz et al., 2015; Bai and Urtasun, 2017; Song et al., 2017). Another approach is using a star-convex polygon representation for cell instances (Weigert et al., 2020), or utilising a vector-field label (Stringer et al., 2020). However, algorithms often lack verification outside of their customised settings. Furthermore, limited programming skills and specific hardware requirements have hindered the deployment of deep learning techniques in practical applications. Therefore, several model architectures and pre-trained models have been made available in user-friendly imaging software such as *ImageJ* (Berg et al., 2019; Stringer et al., 2020; Gómez-de Mariscal et al., 2021) or as cloud-based open-source tools (Lösel et al., 2020; von Chamier et al., 2021). Ideally, this enables users to make predictions on new data without extensive training, hence reducing the need for a large training dataset. Alternatively, the users can train a preconfigured model and fine-tune it using specific data leveraging free cloud GPUs, which reduces hardware requirements on the user side. With the development of new software tools using artificial intelligence, the segmentation of fluorescence microscopy images has made significant progress.

In this work, we explore the applicability of machine learning and artificial intelligence approaches for a fast and reliable model generation of cartilage-like tissue samples from biomedical images. First, we compare classical, machine learning and deep learning approaches for the 3D segmentation of bovine cartilage samples. We benchmark our results against previous results that were obtained by manual segmentation. Subsequently, the impedance and dielectric properties are estimated by finite element simulations using fine-grained models derived from the segmented images. The simulation results serve as another means to compare between simplified ellipsoidal cells and realistic geometries. The effect of uncertainties of the segmentation approach is probed by uncertainty quantification (UQ) and sensitivity analysis (SA). In addition, different samples are considered to elucidate the impact of different tissue volumes, cell distributions, and cell orientations on the computed dielectric properties. Eventually, we discuss how impedance measurements, together with accelerated image segmentation, can contribute to the advancement of biosensor applications in the diagnosis of osteoarthritis. The combination of the biosensor with an electrical stimulation unit has the potential to pave the way for a targeted and personalised treatment of osteoarthritis.

## 2 Materials and methods

### 2.1 Model generation workflow

Confocal microscope images of bovine chondrocytes were employed to generate the geometries and finite element meshes. We used 27 original 16-bit grayscale images of the healthy control samples, obtained from the previous research conducted by Lv et al. (2019). These images have a resolution of 2048 × 2048 pixels per slice with a stack size varied between samples. The voxel size is 0.1099 × 0.1099 × 1 µm. The bovine cartilage explants were dyed with a red fluorescent cell tracker (Red CMTPX Dye, Thermo Fisher), cultured in regular medium for four days prior to imaging, and imaged with a Zeiss LSM510 microscope. The benchmark for cell volume estimation in this study was based on the manually estimated cell volume from the previous study, with a detailed procedure provided in the Supplementary Materials.

#### 2.1.1 Advanced classical image segmentation approach

**FIGURE 1:**
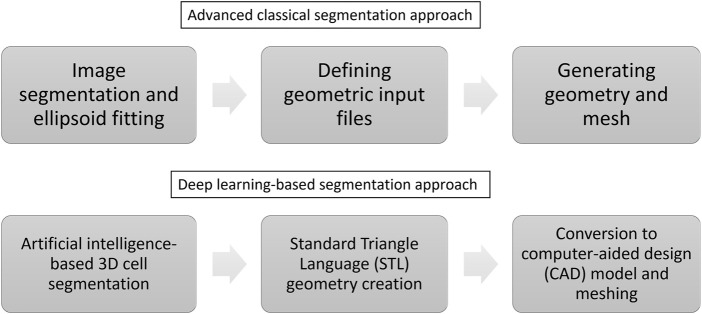
The automation of creating tissue-specific meshes involves three primary stages, utilising advanced classical and deep learning-based segmentation approaches.

The classical automated creation of tissue-specific meshes involves three steps (see Figure 1 and compare (Bennetts et al., 2014)): 1) image segmentation and ellipsoid fitting, 2) defining geometric input files, and 3) generating geometry and mesh.

The geometry and distribution of individual cells can be determined directly from the processed images of specimen-specific tissues. To obtain a geometric description of the cells, we fitted ellipsoids to a contiguous set of voxels associated with each cell using *ImageJ* v.1.53f51 (Schneider et al., 2012)^1^. Eventually, we obtained the cells’ location, radii, and orientation in the tissue. Anisotropic voxel sizes in the 3D images exacerbated the separation of adjacent cells in the z-direction. Therefore, we have resampled the 3D images to obtain image stacks with isotropic voxels. In addition, various filters were employed to reduce noise in the image, enhance contrast, and remove unevenly illuminated backgrounds while preserving low-intensity fine detail. Subsequently, for the sake of reproducibility, an auto-threshold technique was utilised to segment images into cell interior and exterior instead of manually tweaking them. Subsequently, gigantic artifactual objects with an area surpassing 300 μm^2^ were removed from the image slice. Miniature noises and artifacts cannot be filtered out based on this criterion because parts of the cells could be excluded from individual image slices. As an alternative, a volume filter from *BoneJ2* v.7.0.13 (Domander et al., 2021) was employed to remove various tiny noises with a volume of less than 200 µm^3^. Afterwards, holes were filled and touching cells were separated by the *Distance Transform Watershed* algorithm (Legland et al., 2016). Lastly, the cell shape was assumed to be ellipsoidal and the geometric description of the cells was obtained via *3D ellipsoid fitting* (Ollion et al., 2013). The whole procedure was wrapped into an *ImageJ* script to batch process all images.

To define geometric input files, we utilised a format consistent with previous research to store the characteristic dimensions of the ECM and ellipsoids (Bennetts et al., 2014). Due to the fact that each image was captured for only one particular cartilage zone, a geometrical model incorporating multiple cartilage zones can be constructed.

Numerical models such as the finite element method (FEM) require a discretised geometry. For that, we employed the mesh generator *Netgen* (Schöberl, 1997) to automatically construct the geometry. From the geometrical parameter in the input text file, a solid model is assembled using the *Open Cascade Technology (OCCT)* ^2^ geometry kernel, provided in the *Netgen* Python library. Boolean unions of touching objects were implemented to obtain a single cell volume. We meshed the cellular geometries using a pre-determined mesh hypothesis. The meshes were created in such a manner that their surface and volume error were less than 1% with regard to the cell surface area and volume.

#### 2.1.2 Artificial intelligence approaches for 3D cell segmentation

In this work, we suggest the following approach to build a numerical model in an automated manner (see Figure 1): 1) 3D cell segmentation using an artificial intelligence approach on the original images, 2) geometry creation directly from the segmented image using the Standard Triangle Language (STL), 3) conversion to a computer-aided design (CAD) model and meshing.

Before finding a suitable approach, we compared various methods, namely, the Trainable Weka Segmentation (TWS) method (Arganda-Carreras et al., 2017), *DeepImageJ* (Gómez-de Mariscal et al., 2021), *Biomedisa* (Lösel et al., 2020) and *ZeroCostDL4Mic* (von Chamier et al., 2021). Unfortunately, we could not achieve satisfactory results using these methods. A detailed description of the procedure that we followed for each approach is reported in the Supplementary Material.

We chose *Stardist* because it was developed with a focus on blob-shaped cells in a crowded environment (Weigert et al., 2020). *StarDist* uses a slightly modified variant of 3D U-Net as a neural network backbone to predict the radial distances (star-convex polyhedron representation) and object probabilities (indication of how likely a pixel is part of an object). There are only a few hyperparameters in *StarDist* to be tuned. We trained the model on three different platforms to assess hardware configuration requirements for deep learning model training. A workstation with Intel Core i7-10700 (64 GB RAM) without GPU support, and the post-processing node of the HAUMEA HPC cluster at the University of Rostock with a GeForce179 RTX 2080 Ti GPU (11 GB VRAM) and an Intel Xeon Gold 6234 CPU (8 cores, 16 threads) with 1.5 TB RAM were utilised for training the deep learning model.

Because the model input is the original image, no pre-processing step is required. We used the fitted-ellipsoid images as ground truth. These images were generated by fitting ellipsoids to the voxels of the cells using the *3D ellipsoid fitting* plugin in *ImageJ*. This process was performed after applying the *Distance transform watershed*. The fitted ellipsoid images are represented as 16-bit images, where each ellipsoid is assigned a unique voxel intensity value. These intensity values serve to distinguish individual cells within the image. An example of a slice in a training pair can be seen in Supplementary Figure S10. To increase the diversity of the training data and improve the models’ performance, we applied a basic data augmentation comprising a lateral flip, rotation, and random intensity scaling. We randomly selected 23 images for training/validation and four images for testing the deep learning model. The test images were labelled according to their source, sample number and cartilage zone, such as animal 1 sample 1 top zone (A1S1), animal 2 sample 1 top zone (A2S1), animal 4 sample 1 deep zone (A4S1) and animal 5 sample 2 middle zone (A5S2). The testing data set includes one good-quality image (A1S1), two noisy images (A2S1 and A5S2) and one very noisy image (A4S1). As a precaution against memory issues, the training/validation dataset has been cropped into 92 equal XY-size images of 512 × 512 pixels (78 for training and 14 for validating). In total, we have 6 GB of data.

To assess the model performance, we used the accuracy estimate introduced by Weigert et al. (2020) as

$$accuracy\left(\tau \right)=\frac{TP}{TP+FN+FP}$$ (1)

where *τ* is the overlap threshold, true positives TP are the number of correct predictions, unmatched predicted instances are FP false positives, and unmatched groundtruth instances are identified as FN false negatives. A predicted object *I*_pred_ and ground-truth object *I*_gt_ are considered as one correct prediction if they have an intersection over union (*IoU*) that fulfils

$$IoU=\frac{I_{\text{pred}}\cap I_{\text{gt}}}{I_{\text{pred}}\cup I_{\text{gt}}}\geq \tau \;.$$ (2)

We also report *precision* (see Eq. 3), *recall* (see Eq. 4) and *F*1 − *score*, also known as the Dice score (see Eq. 5). They are commonly employed in practice to access the model performance (Laine et al., 2021). *Precision* measures the proportion of predicted voxels in the segmentation results that match the ground truth voxels. Over-segmentation results in lower precision scores. On the other hand, *recall* represents the proportion of predicted voxels in the ground truth that were correctly identified. Low recall scores are caused by under-segmentation. The *F*1—*score*, which combines precision and recall, provides an overall assessment of segmentation performance. An increase in *F*1, indicates better segmentation performance, with a value closer to 1 indicating more accurate and precise segmentation.

$$precision=\frac{TP}{TP+FP}$$ (3)

$$recall=\frac{TP}{TP+FN}$$ (4)

$$F1=2\times \frac{precision\times recall}{precision+recall}$$ (5)

Apart from building the model from scratch, we also employed transfer learning techniques with the *StarDist* model. Our approach involved retraining the pre-trained *StarDist* model from Rasse et al. (2020),^3^ with our specific data. The pre-trained model had been previously trained on an *Arabidopsis thaliana* lateral root nuclei dataset (Wolny et al., 2020). Furthermore, for comparison, we considered *ilastik* (Berg et al., 2019) and *Cellpose3D* (Eschweiler et al., 2022). A comprehensive description of the methodology employed in implementing *ilastik* and *Cellpose3D* can be found in the Supplementary Material.

For the geometry creation, we generated the STL mesh of the cells directly from the deep learning predicted images rather than fitting the ellipsoid to derive the characteristic dimensions of the cells. *scikit-image* ^4^ was leveraged to accomplish this task using the marching cubes algorithm (Lewiner et al., 2003). To improve the STL mesh quality, an original surface representation was remeshed and smoothened employing isotropic explicit remeshing (Peercy, 1993) and Taubin smoothing (Taubin, 1995) available in PyMeshLab (Muntoni and Cignoni, 2021). A simplification of the resulting mesh can then be achieved using the Quadric Edge Collapse Decimation technique (Garland and Heckbert, 1997). Subsequently, the simplified and smoothed STL meshes were converted to CAD models using *FreeCAD* ^5^. During this step, the common parts of the cell membrane between two intersecting cells are removed by the conversion algorithm, i.e., the cell cytoplasms are not separated by a membrane. The CAD geometries were, thereafter, utilised to generate quality tetrahedral volume meshes in *Netgen*.

### 2.2 Numerical modelling

Many therapeutic procedures associated with the electrical stimulation of biological samples involve slow variations in electromagnetic fields (van Rienen et al., 2005). Under the assumption that magnetic fields and eddy currents are negligible, the electro-quasistatic (EQS) potential *ϕ* can be obtained by solving

$$\nabla \cdot \left[\left(\sigma +j\omega \varepsilon \right)\nabla \phi \right]=0\;,$$ (6)

where *σ* is the conductivity, *ɛ* = *ɛ*_*r*_ *ɛ*_0_ is the permittivity with *ɛ*_0_ = 8.854 × 10^-12^ F m^−1^, and *ω* is the angular frequency. We solved Equation (6) by the finite element method (FEM) with second-order curved elements in *NGSolve* (Schöberl, 2014). As the cell membrane is very thin compared to the remaining components of the geometry, we used a thin layer approximation to describe it with a thickness of 7 nm (Pucihar et al., 2006; Zimmermann, 2022). For the dielectric properties of the cell’s components, a subscript *m* is a cell (plasma) membrane, *cyt* is a cytoplasm and *buf* is the cell culture medium. We selected *σ*_*m*_ = 8.7 × 10^−6^ S m^−1^, *σ*_*cyt*_ = 0.48 S m^−1^, *σ*_*buf*_ = 1 S m^−1^, $\varepsilon_{r}^{m}=$ 5.8, $\varepsilon_{r}^{\mathrm{cyt}}=60$ and $\varepsilon_{r}^{\mathrm{buf}}=80$ (Ermolina et al., 2000; Zimmermann, 2022). A voltage drop of 1 V was imposed across the simulation domain. Frequencies from 1 kHz to 1 THz with ten logarithmically spaced points per decade were considered. The Conjugate Orthogonal Conjugate Gradient (COCG) solver with a Jacobi preconditioner was used to solve the arising linear system of equations. The impedance *Z* of the considered samples was computed from the solution of Equation (6) as described in (Zimmermann et al., 2021). The dielectric properties were estimated from the impedance for a known electrode geometry (Zimmermann and van Rienen, 2021) by employing the open-source package *ImpedanceFitter* (Zimmermann and Thiele, 2021). All simulations were carried out on the high-performance computing (HPC) cluster HAUMEA of the University of Rostock (each computing node equipped with 2 Intel Xeon Gold 6,248 CPUs with in total 40 cores and 192 GB RAM). Unless otherwise stated, we used the message passing interface (MPI) to parallelise the simulations across multiple nodes. The interested reader can find more details elsewhere (Zimmermann, 2022).

### 2.3 Uncertainty quantification

We addressed potential inaccuracies of the deep learning prediction by Uncertainty Quantification (UQ) techniques. We considered two cases.

In case 1, an error in the cell’s location, volume, or orientation was covered. This case primarily aims to determine which error from the geometrical variation above significantly impacts the numerically computed impedance and dielectric properties. For that, we used a geometry constructed from one cell exported from the CAD model derived from the deep learning-predicted image. To examine the mutual cell interaction in the first UQ approach, the cell was placed in the centre of a square box and could move in the xyz-direction, while its copy was fixed at the top right of the box. The box volume was chosen in such a way that the volume ratio is similar to the value of the original volumetric images. The fixed cell’s position was chosen so that it could not intersect with the movable cell. An illustration of different cell positions, volumes and angles can be seen in Supplementary Figure S11.

In case 2, uncertainties of the cell geometry were not considered but the influence of an uncertain membrane thickness and uncertain dielectric properties were probed. Here, we used the deep-learning-based geometry of A4S1 to test the approach on a realistic geometry.

All potential error sources and assumed hypotheses in the UQ analysis are summarised in Supplementary Table S3. To keep the computational amount reasonably small, we employed polynomial chaos expansion (PCE), being much more efficient than the Monte Carlo approach (Tennøe et al., 2018). For example, case 1 with five uncertain parameters required 254 FEM model evaluations with the PCE method while case 2 required 662 model evaluations. We used a modified version^6^ of *Uncertainpy* (Tennøe et al., 2018). The expansion coefficients were estimated based on a point collocation method with the polynomial order of four as suggested in (Eck et al., 2015). To obtain the 5th and 95th percentile, 10^4^ samples were drawn from the PCE surrogate model.

## 3 Results

### 3.1 Image segmentation and mesh generation

#### 3.1.1 Advanced classical segmentation approach

**FIGURE 2:**
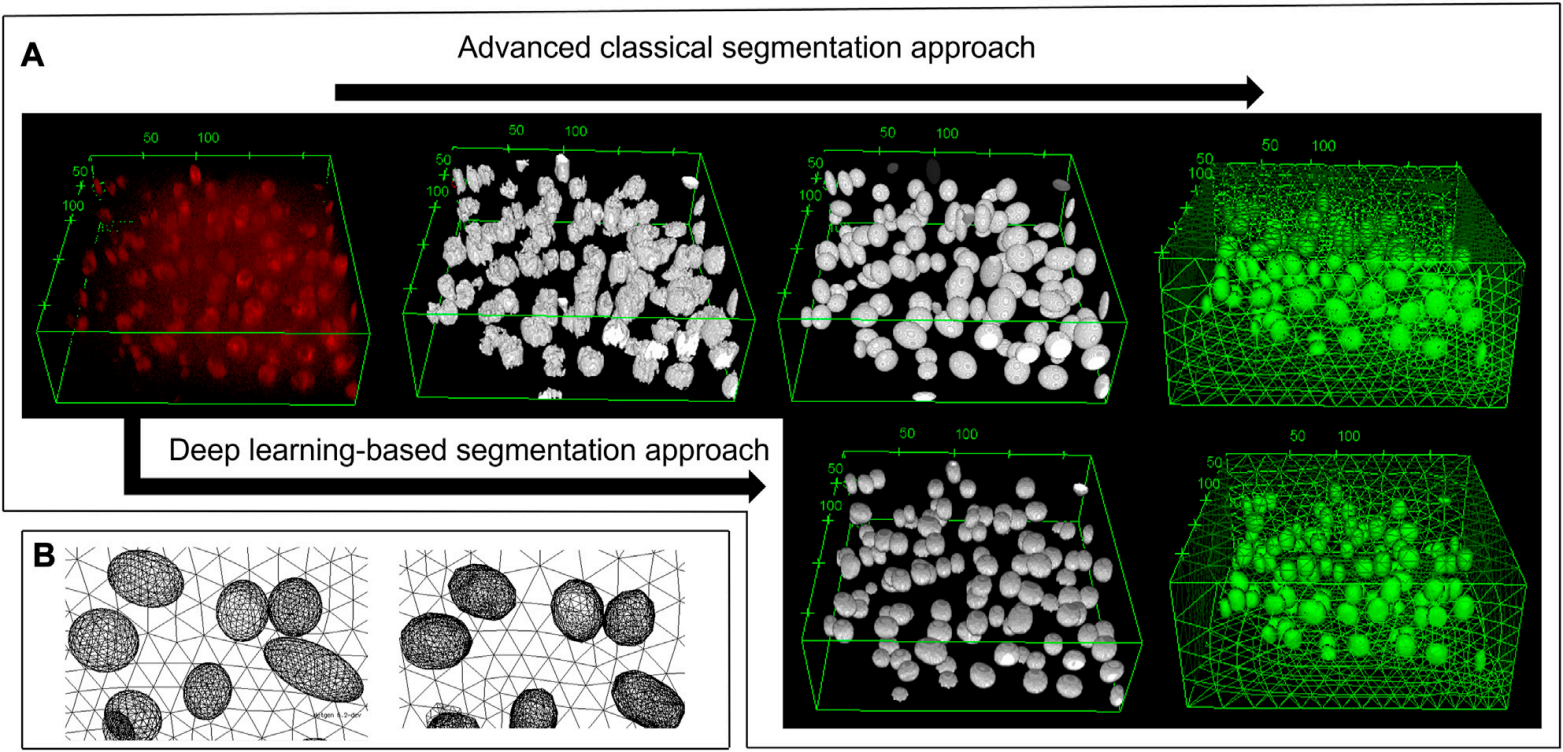
3D view of the deep zone from animal 4 sample 1. **(A)** From top left to top right: the original image, the pre-processing image, the fitted ellipsoid, and the mesh based on the advanced classical segmentation approach. Bottom left and bottom right: the deep learning prediction and its mesh, respectively. Using the deep learning-based approach, one can obtain the segmented image without any pre-processing steps. **(B)** The zoomed-in image of the mesh from fitted ellipsoid (left) and the deep learning predicted image (right). As being constructed from several triangles of the STL geometry, the deep learning mesh was not smooth in contrast to the ellipsoid mesh. Most of the cells can be predicted, however there are some volume and position errors. The unit of the specified lengths is µm.

Using 27 images from different cartilage zones, the classical segmentation approach together with the ellipsoidal fitting yielded an average cell volume of 2,157 ± 957 µm^3^. This cell volume is consistent with the reported value of 2,270 ± 590 µm^3^ by Lv et al. (2019), which we used as a benchmark. It indicates that the segmentation process is reliable. Furthermore, the obtained geometric model comprising the fitted ellipsoids resembled the original microscope image very well (see example in Figure 2).

However, some of the processed geometries could not be meshed. We identified small voids between intersecting or touching cells as the culprit. The error could be fixed by adding small spheres to cover the void. For that, an initial intersection check was performed on all objects and then an indexed list of the touching objects was created. The indices were mapped to a graph employing *NetworkX* (Hagberg et al., 2008)^7^. The nodes corresponding to intersecting objects were connected. In this way, cycles (loops) within the graph could be detected. Whenever a cycle in the graph is detected, the common volumes between the individual cells are computed and fused. In the case of a void, the fused common volumes do not form one single solid. The located void spaces were filled with a sphere.

#### 3.1.2 Deep learning-based 3D cell segmentation approach

**FIGURE 3:**
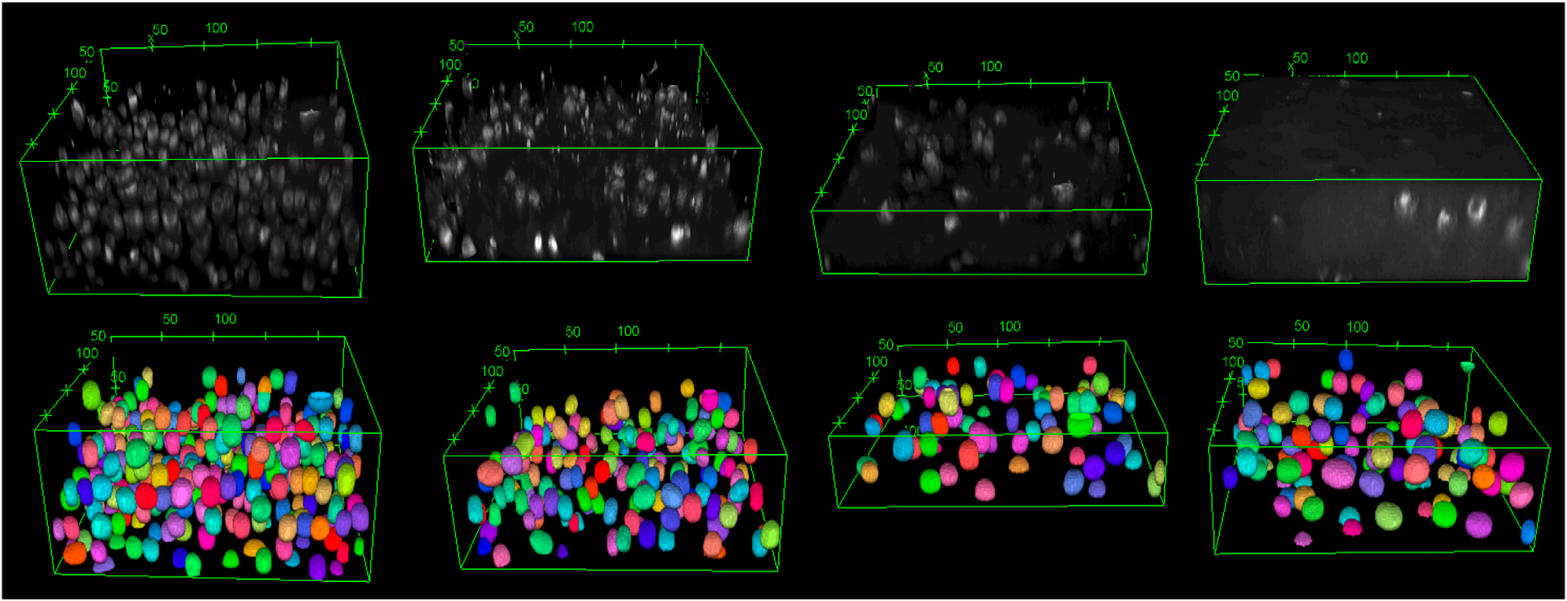
The original images are shown from top left to top right: good-quality image, two noisy images, and a highly noisy image. The corresponding deep learning predictions of the original image are shown from bottom left to right. The length unit in the images is µm. The deep learning model demonstrated good performance not only on high-quality images but also on noisy images. As a 3D visualisation of the highly noisy images is difficult, different slices from the original image and its corresponding deep learning prediction are shown in Figure 4.

**FIGURE 4:**
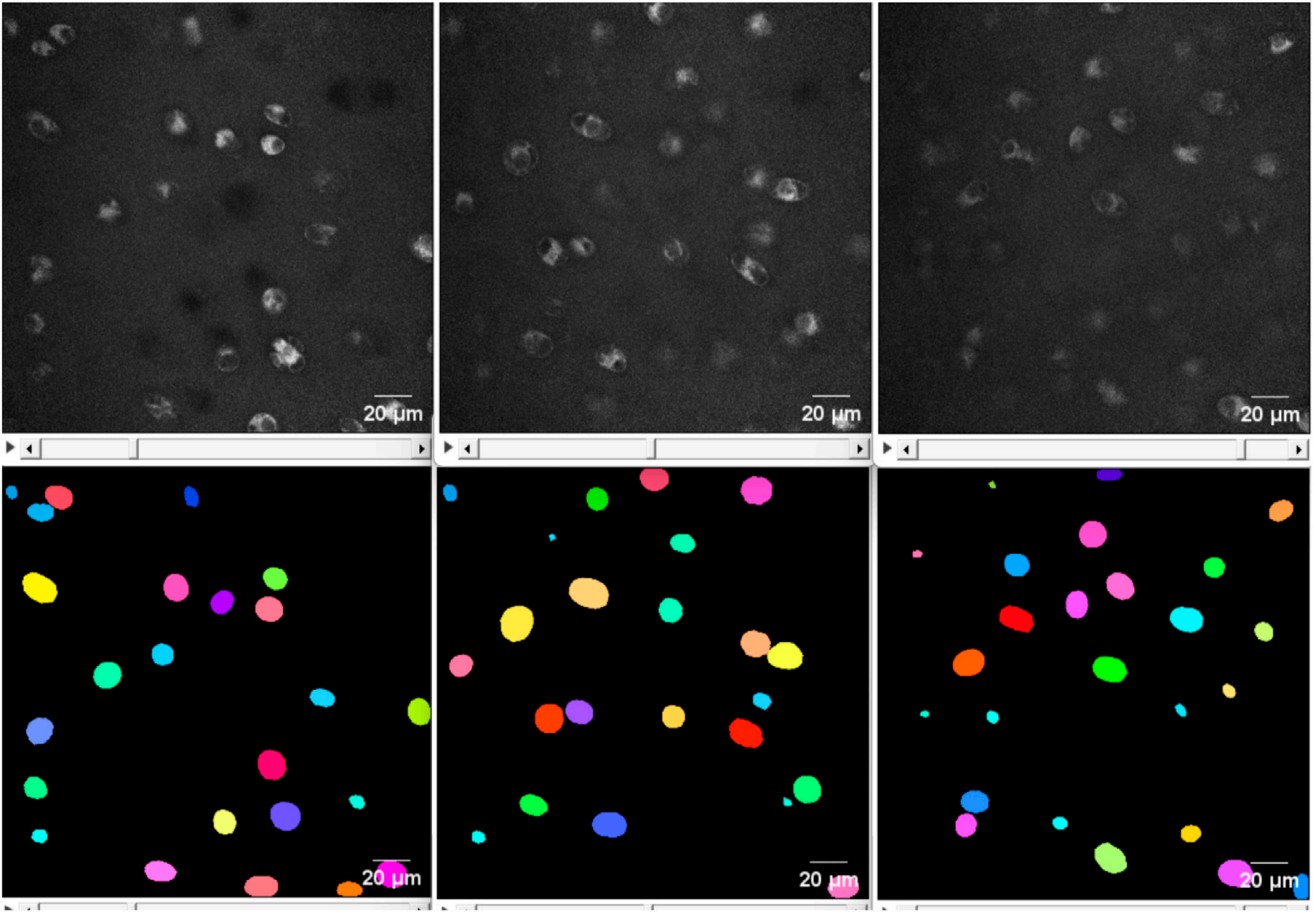
From top left to top right: The 2D view of slice 20, 37 and 70 from the original image of the highly noisy image (see Figure 3). From bottom left to right: the corresponding deep learning predictions using *StarDist* of the above slices from the original image. The deep learning model evidently demonstrated good performance on even highly noisy images.

The training of *StarDist* on our workstation without GPU support could not finish due to memory issues. Still, it succeeded on the HPC post-processing node after around five hours for 400 epochs when the loss value had converged. Subsequently, the trained deep learning model was validated with test images of varying quality. For that, we chose a good-quality image, two noisy images, and one highly noisy image. The predictions of the deep learning model, which was trained on our data, are shown in Figures 3, 4. The results suggest that the deep learning model performed well not only on high-quality images but also on noisy and highly noisy images. The *precision*, *recall* and *F*1 score with a recommended *IoU* threshold *τ* of 0.5 on the validation and test data, given in Supplementary Table S2, indicated that no over- or under-segmentation could be observed as those performance metrics are larger than 0.7. The evaluation metrics at different *IoU* threshold are provided in Supplementary Figure S4, S5. Moreover, the model achieved a test accuracy of approximately 80%, while the validation accuracy was around 60%. This result is in contrast to the general expectation that the performance metrics of the validation should be higher than those values from test data. We believe that our result can be explained by the data quality. In the cropped images, which we used for validation, several objects touched the image boundary. *StarDist* cannot detect objects in contact with the boundary of the images well, which could explain the relatively low validation accuracy. In practice, the deep learning model achieved a good prediction despite the relatively low validation accuracy.

To underline this point, we considered the total number of cells and the cell volume as additional model evaluation metrics. The predicted cell volumes did not deviate significantly from those obtained from the classical ellipsoid-fitting approach (see Table 1 for a comparison of the obtained volume ratios). Nonetheless, the number of cells counted using the deep learning method was 9.5% lower than in the classical approach. Moreover, a visual comparison of expected output versus deep learning prediction (refer to Supplementary Figure S6) also shows that most of the cells can be detected but some of them have slightly smaller volume than the cell in the ground truth. Using four test images, the classical approach detected 748 cells with an average volume of 1,714 ± 1,180 μm^3^, while the deep learning method identified 683 cells with an average volume of 1,601 ± 620 µm^3^. It is worth noting that the average cell volume is smaller than the reported value for all samples due to the predominant presence of cells from the top zone in the test image. Typically, chondrocytes in the top zone have the smallest volume compared to cells in other zones.

**TABLE 1:** The volume ratio of different sample geometries obtained from deep learning and the classical fitted ellipsoid approach. The deep learning geometry is the CAD model converted from the simplified and smoothened STL geometry. The number of faces for both the original STL geometry and its simplified and smoothened version also given.

Volume ratio/%				
Sample	Initial STL faces	Simplified and smoothed STL faces	Deep learning geometry	Fitted ellipsoid geometry
A1S1	4,967,008	42,960	4.3	4.8
A2S1	2,785,728	25,304	3.0	3.2
A4S1	2,422,692	19,848	2.9	3.6
A5S2	1,411,760	12,590	2.1	2.8

**FIGURE 5:**
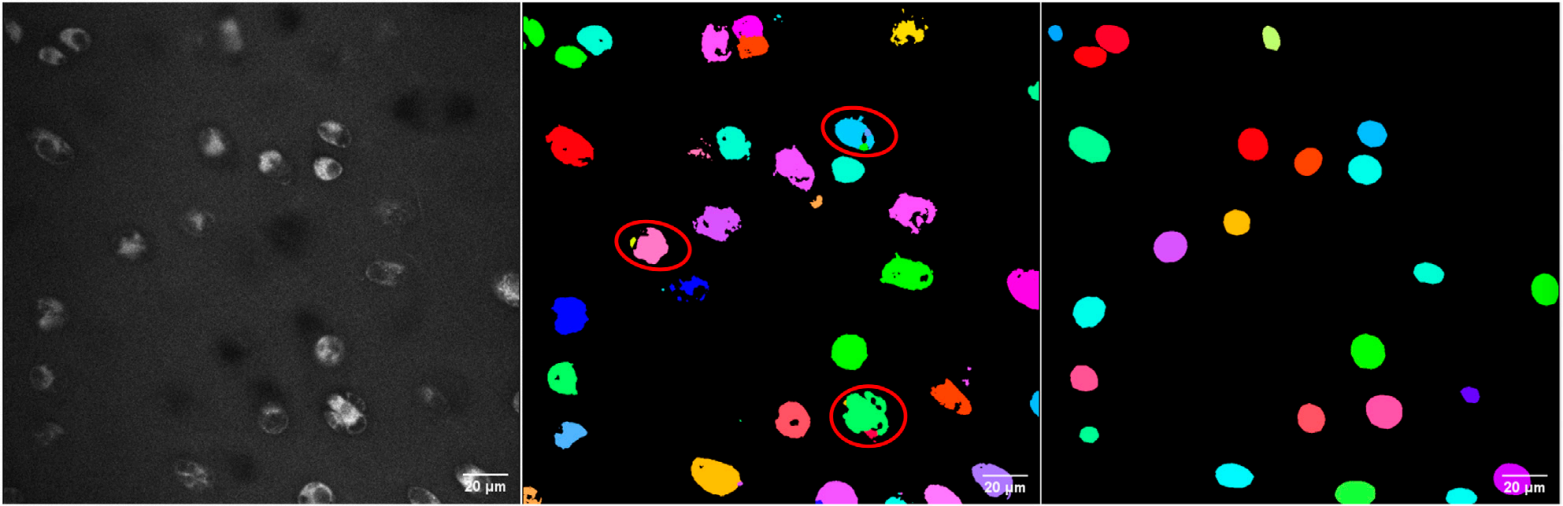
2D view of the middle slice from a noisy image. From left to right: the original image, the result of the *ilastik* segmentation and the result of *StarDist*. Using the *ilastik* model, some parts belonging to an individual cell were detected as different objects (indicated with the red ellipses), leading to more cells and a smaller average cell volume. Furthermore, it is required to perform post-processing such as additional filling holes, dilation and erosion to make the cell boundary complete and smoother before converting to STL geometry.

The *ilastik* model predicted 1,050 cells with an average volume of 1,487 ± 1,343 μm^3^, resulting in a 28% increase in the number of cells compared to the classical approach. As can be observed from Figure 5, utilising the *ilastik* model resulted in the detection of certain cell parts as separate objects, leading to an increased number of cells and smaller average cell volume. The *StarDist* prediction demonstrated better concordance with the classical approach, as evidenced by a smaller discrepancy in both the number of cells detected and their average volume. Moreover, due to the rugged shapes of the cells segmented using *ilastik*, it is necessary to apply post-processing techniques such as hole-filling, dilation, and erosion to obtain smooth and complete cell borders before converting them into STL geometry. Hence, *StarDist* is the method of choice in our specific case.

With *CellPose3D*, all four predicted images showed only a black background instead of cells (see Supplementary Figure S2). Due to the significant increase in training and prediction times compared to *StarDist* (approximately five times for training and eight times for prediction), we did not conduct further investigation into the algorithm failure in our case. It is worth noting that *StarDist*, unlike *CellPose3D*, does not require a data preparation step and uses the original format of the images as input. *StarDist* appears to be a more suitable algorithm for our specific application.

In order to better assess the performance of different segmentation approaches, we analyzed the distribution of cell volumes (Supplementary Figure S3). A few artifacts having a volume greater than 5,000 µm^3^ were identified as cells by the advanced classical approach. These artifacts shared the same pixel intensity as cells and could not be separated by means of the *Distance Transform Watershed* algorithms. Consequently, based on their fitted ellipsoid volume, these artifacts were excluded from the final geometry during the defining geometry input step. On the other hand, when utilising *StarDist*, all cell volumes fell within a reasonable range, specifically smaller than 4,000 µm^3^. It appears that *StarDist* can avoid wrong predictions of objects that deviate significantly in size from the training data. However, this characteristic may also be viewed as a disadvantage, because it necessitates ensuring that new images have a similar pixel size to the training images. Lastly, as previously mentioned, the usage of *ilastik* resulted in the detection of certain cell components as distinct objects. Hence, this led to an increased count of cells and a smaller average cell volume.

Regarding the model configuration of *StarDist*, we followed the advise by the developer. The training patch size was set to (16, 512, 512) to guarantee that it is smaller or equal to the size of the smallest annotated training image. Additionally, the grid parameter of (1,4,4) was used to specify the maximal size of well-segmented objects based on the neural network’s receptive field. The training batch size could not be set greater than one due to a lack of memory. A flexible parameter in the fitting procedure is the number of rays. We found that an adequately accurate reconstruction of the 3D cell in ground-truth images can be acquired with 96 rays. The evaluation metrics of the model did not improve when the number of rays was increased to 128. An even higher number of rays led to increased memory consumption that was beyond the capabilities of our systems. Moreover, we tuned the learning rate in conjunction with different model depths. The detailed results are provided in Supplementary Table S1. Based on our analysis, increasing the model depth to four resulted in memory issues. Additionally, when using a learning rate of 0.1 and 0.01, the model did not show any improvement as the loss fluctuated and remained relatively constant (refer to Supplementary Figure S7, S8). We observed that the model achieved the best performance with a learning rate of 0.0003 and a U-Net depth of two. As can be seen from Supplementary Table S1, reducing the learning rate to 0.0001 gained 1% in validation accuracy, but the training accuracy was reduced by 3%. Moreover, the model trained with a learning rate of 0.0003 achieved a lower training loss (0.5042) compared to the model trained with a learning rate of 0.0001 (0.5576) while the validation loss remained almost the same (0.6744 and 0.6713). Therefore, the learning rate of 0.0003 performed better in terms of training and is the value of choice. Our selection was further confirmed by the testing accuracy: 80% with the learning rate of 0.0003% and 77% with the learning rate of 0.0001.

By initialising the model with pre-trained weights from the *StarDist* model Rasse et al. (2020) along with the optimal hyperparameters mentioned earlier, all the accuracies of the models were reduced. Specifically, the training, validation, and test accuracies were approximately 2%, 3%, and 4% lower than the values when training the model from scratch. This decline in performance can be attributed to the significant influence of initial weights on the convergence of neural networks. Ill-suited weight initialisation might hinder the model’s ability to adapt and learn effectively from the new dataset (Glorot and Bengio, 2010; Sutskever et al., 2013).

We chose fitted ellipsoid images as the ground truth data because they were in good agreement with the manual segmentation (see Supplementary Figure S9) and *StarDist* is specialised for objects with blob-like characteristics. Alternatively, we used the images after applying the auto-threshold (see an example in Supplementary Figure S1) as ground truth. However, they produced poor predictions because the cells could have holes, rugged shapes or were incomplete close to the image boundary because several cells were not dyed completely. The training in *StarDist* involves fitting the ground-truth labels with star-convex polyhedra. Eventually, we concluded that the deep learning-based segmentation using *StarDist* yielded sufficiently good results to prepare geometries for the FEM simulations. Strikingly, the deep learning prediction using the trained model took less than three minutes on a standard workstation while the advanced classical technique, including the pre-processing and ellipsoidal fitting, took approximately 15 minutes to process one image. By utilising the HAUMEA HPC cluster’s post-processing node, the prediction time for the deep learning method was reduced to less than one minute, whereas the classical method required seven minutes to process one image.

#### 3.1.3 Generating FEM mesh based on deep learning predictions

The STL geometries generated from deep-learning-predicted images maintained the total volume and surface area with errors of less than 0.1% (Table 2). However, simplification is necessary to convert the generated STL meshes into CAD models. Table 1 provides the number of faces for the original STL geometry, as well as its simplified and smoothened version. The initial STL geometries were remeshed to generate an isotropic mesh. This process maintains the original volume and surface area. To ensure successful tetrahedral meshing, the obtained meshes were subjected to smoothening and subsequently simplified. Smoothing primarily contributed to a reduction in the surface area, while simplification was mainly responsible for a decrease in volume. As shown in Table 2, the final CAD models’ total cell area and volume decreased by approximately 15% and 5% with respect to those values from the deep learning predicted images. Smoothening the stepped structure of the STL geometry and the shape edges at the top and bottom of the cells due to the reconstruction of the marching cube algorithm from 3D stack images led to a large surface reduction. Nonetheless, the smoothening and simplification steps are inevitable to achieve FEM meshes.

**TABLE 2:** The total cell volume and surface area from deep-learning predicted images, the relative difference in total cell volume and area from the STL geometry generated from deep learning prediction and the final CAD geometry.

Sample	Deep learning predicted image		Original STL geometry Relative difference/%		CAD geometry Relative difference/%	
	Volume/µm^3^	Surface area/µm^2^	in volume	in surface area	in volume	in surface area
A1S1	462,210	232,446	0.07	0.04	5.94	14.91
A2S1	257,590	129,761	0.07	0.04	4.93	14.41
A4S1	234,963	104,809	0.07	0.04	4.24	15.78
A5S2	128,271	63,716	0.14	0.05	5.25	15.59

**FIGURE 6:**
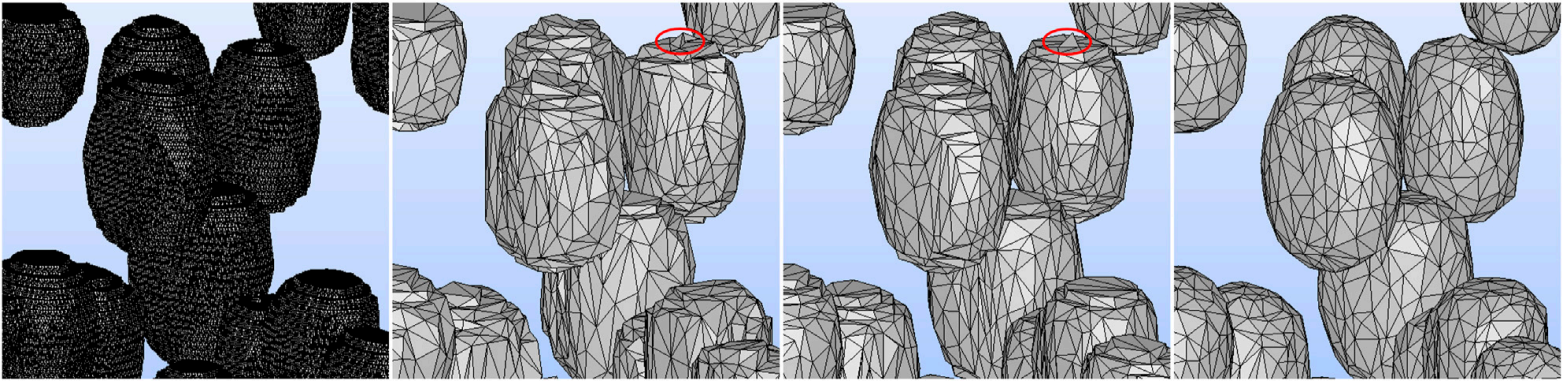
From left to right: The zoomed-in images of the A5S2 STL geometry generated from the voxel size of the deep learning predicted image with 1,411,760 faces, the simplified geometry employing two steps smoothing, Laplacian smoothing and Taubin smoothing. After smoothing and simplifying, the STL geometry has 12,590 faces. The Taubin algorithm produced the smoothest cell surface, facilitating FEM meshing. In the middle panels, the mesh failure’s culprits are visible: the edges at the top of the cell, which are marked by the red ellipse.

We tested three different smoothing techniques in PyMeshLab, including Laplacian smoothing (surface preserving), Taubin smoothing, and two steps smoothing that included normal smoothing and vertex reposition. Among these techniques, the Taubin algorithm was the most suitable for preserving the cell volume. Crucially, it is the only technique that allows for successful FEM meshing. Moreover, two simplification methods available in PyMeshLab, edge collapse for marching cube meshes and quadric edge collapse decimation, were evaluated. The latter was found to be more effective in preserving the original surface area and volume of the STL mesh. The smoothened and simplified STL geometries using various smoothing techniques are illustrated in Figure 6.

The convergence of FEM meshes generated from deep-learning-based geometries was observed as the numerical simulation produced consistent results (with relative differences less than 0.05% as shown in Supplementary Figure S20) upon increasing the number of elements in the mesh through the use of smaller mesh sizes. The time required to convert the original STL generated from deep learning prediction to a CAD model is approximately five minutes while creating a CAD model from ellipsoid parameters derived from the classical segmentation approach takes less than one minute. Thus, using a deep learning approach on a standard workstation, creating a CAD model from the original image takes about eight minutes, whereas using the classical segmentation approach takes around 16 minutes.

### 3.2 Numerically estimated dielectric properties

Numerical models were generated from the four samples that were used to test the trained deep learning model in the previous section. The models are named after their source and sample number and were chosen from different cartilage zones, i.e., animal 1 sample 1 top zone (A1S1), animal 2 sample 1 top zone (A2S1), animal 4 sample 1 deep zone (A4S1) and animal 5 sample 2 middle zone (A5S2). For every sample, two models were generated: one using the deep learning geometry and the other one using the geometry obtained by ellipsoid fitting.

**FIGURE 7:**
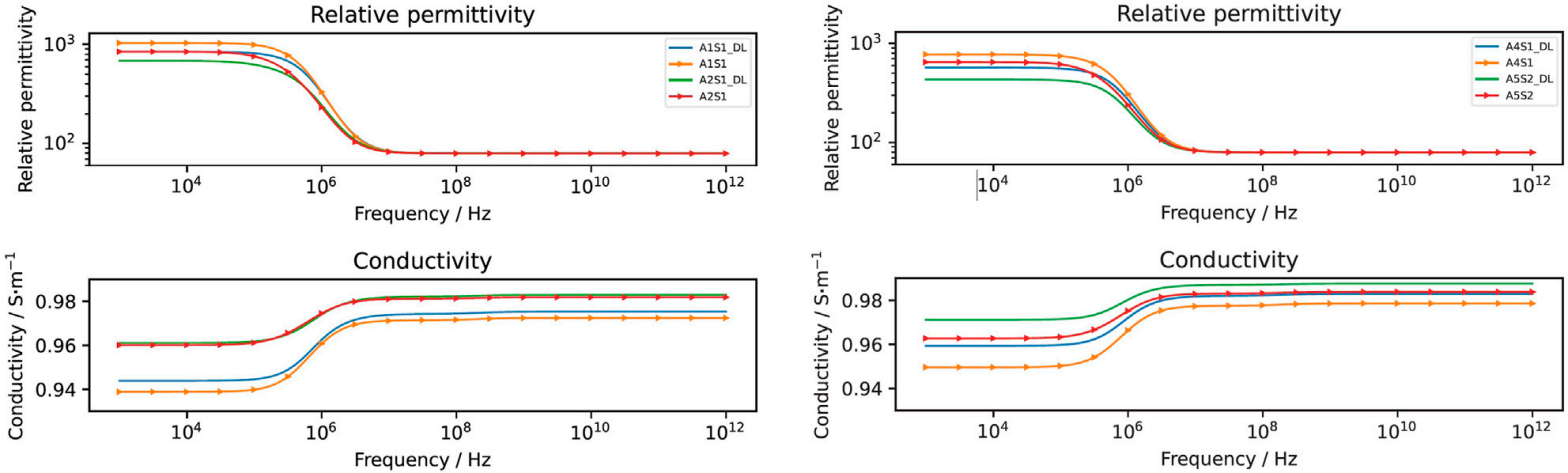
The computed dielectric properties using the deep learning approach (straight line and denoted by DL) and the classical approach (line with markers). The relative difference between the results obtained by the two approaches is shown in Supplementary Figure S19.

**FIGURE 8:**
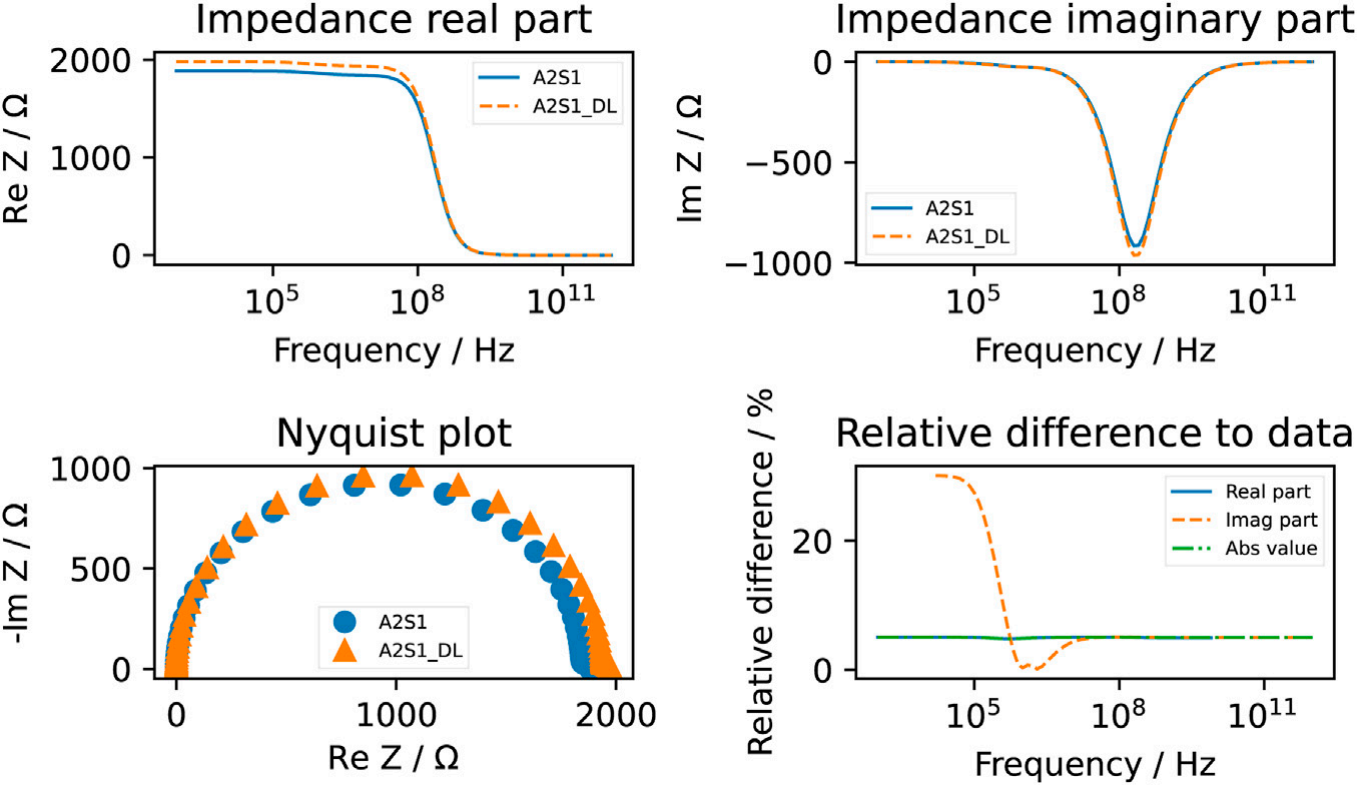
The computed impedance of the A2S1 sample using the deep learning approach (dashed line and denoted by DL) and the classical approach (straight line). The relative difference of the real part of the impedance was around 5%. Note that the relative difference of the imaginary part is not always computed because it tends to approach zero at lower frequencies. Below 1 MHz, an apparent deviation between the two computed imaginary parts can be observed.

The computed conductivity and relative permittivity using different segmentation methods are shown in Figure 7 and in Table 3. Additionally, Supplementary Figure S19 illustrates the relative difference in computed dielectric properties for each sample. All samples reveal a dispersion around 1 MHz, which is expressed by an increase of the conductivity and an increase of the permittivity. The dispersion in this frequency range is called *β*-dispersion and is a consequence of interfacial polarisation at the cell membrane (Kuang and Nelson, 1998). A small dispersion can be observed between 100 MHz and 1 GHz. The permittivity approaches a fixed value at high frequencies for all samples, which indicates that the cells do not have an impact in this frequency region. At frequencies below 1 MHz, differences between the individual samples but also between the segmentation methods can be observed. We observed that irrespective of the selected segmentation method, a higher volume ratio leads to a larger permittivity and a lower conductivity. This result is in alignment with analytical equations describing the dielectric properties of cell suspensions (Asami, 2002). The difference in the volume ratio also explains the deviations with regard to the segmentation method. While the relative difference between the results obtained by the two segmentation methods of the permittivity is approximately 20%–35%, the relative difference in the conductivity is always below 1%. The reason for this observation lies in the small variation of the conductivity. The conductivity is dominated by the conductivity of the extracellular medium but not crucially influenced by the cells (Zimmermann, 2022). A better indicator for the comparison of the segmentation methods is the impedance. At frequencies below 1 MHz, the impedance is mostly real-valued (see an example in Figure 8). The relative difference of the real part of the impedance was for all samples about 5%–10% (Supplementary Figure S16, S17, S18). This is above the measurement resolution of a high-resolution impedance spectrometer and could thus be in general measured.

**TABLE 3:** Relative difference in the computed relative permittivity and conductivity from deep learning mesh and fitted ellipsoid mesh concerning total cell volume.

Sample	Total cell volume/µm^3^			Maximum relative difference/%	
	Deep learning geometry	Fitted ellipsoid geometry	Relative difference/%	Relative permittivity	Conductivity
A1S1	434,741	487,942	10.9	18.4	0.5
A2S1	244,898	257,139	4.8	19.2	0.1
A4S1	224,994	280,629	19.8	26.4	1.0
A5S2	121,793	160,341	24.0	33.3	0.9

As the focus of this work is on the use of deep learning approaches for the model generation, we paid special attention to the complexity of the model generation. The classical segmentation approach has significant disadvantages regarding the meshing workflow. In particular, the intersection of objects has to be checked one-by-one, which is a time-consuming task. For example, checking 500 cells necessitates 124,750 checks. Moreover, the geometrical representation of intersecting cells can require edges that are much smaller than the characteristic size of the model. Together with the occurrence of small voids between touching cells, the classical segmentation approach can lead to low-quality mesh elements or even a failure in the meshing process. The suggested fix by filling the void with spheres requires a careful choice of the radius and possible manual interaction. A low-quality mesh usually leads to a slow convergence of the FEM solver. Furthermore, we experienced problems with generating a distributed mesh when low-quality elements were present. A distributed mesh is required for an MPI-parallel run on the HPC cluster. As the low-quality elements are linked to the cell geometry, we could not further improve the mesh quality of the A2S1 sample generated by the classical approach. Consequently, we could not run the simulation using MPI but had to resort to a shared-memory run on a single computation node. Hence, computing the dielectric properties on this mesh took more than five hours for 2,030,984 degrees of freedom (DOFs). On the other hand, the deep learning-based mesh generation did not suffer from this drawback as a high-quality mesh could always be obtained from the STL mesh describing the cell surfaces. As a result, all computations could be conducted using MPI, which significantly reduces the computational time compared to the shared-memory computation (see details in Table 4). For example, models with a similar number of DOFs could be solved in a couple of minutes.

**TABLE 4:** The number of degrees of freedom (DOFs) and the computational time of different sample geometries obtained from deep learning and the classical fitted ellipsoid approach.

Sample	DOFs		Computational time/minute	
	Deep learning geometry	Fitted ellipsoid geometry	Deep learning geometry	Fitted ellipsoid geometry
A1S1	3,863,123	2,289,160	18.2	8.8
A2S1	3,301,823	2,030,984	8.2	314
A4S1	2,655,629	2,625,668	6.9	7.5
A5S2	1,705,226	748,175	3.5	1.5

### 3.3 Uncertainty quantification and sensitivity analysis

**FIGURE 9:**
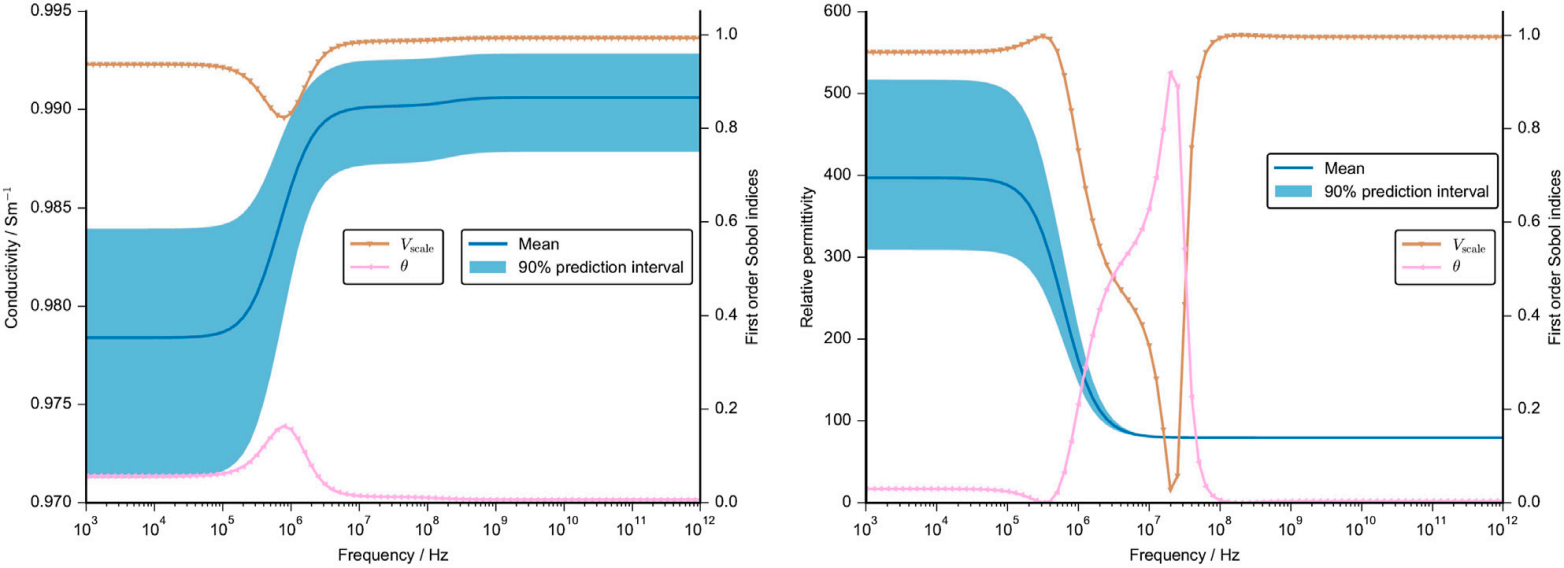
Mean value and 90% prediction interval of the conductivity (left) and the permittivity (right) are demonstrated over a frequency range. The frequency-dependent first order Sobol indices are presented for parameters with a Sobol index of more than 0.05. The assumptions for the UQ analysis of the geometrical parameter, namely, x, y, z position, volume and cell angle, are given in Supplementary Table S3. Note that the other cellular dielectric properties were set at a constant.

We performed a UQ analysis with regard to geometrical uncertainties (case 1, compare Section 2) in a unit-cell geometry. The mean conductivity and permittivity of this configuration together with the 90% prediction interval are shown in Figure 9. The results are shown together with the first order Sobol indices, which measure the individual contribution of an uncertain parameter (Tennøe et al., 2018). We excluded parameters that did not have a Sobol index greater than 0.05 over the entire frequency range. Based on the excluded parameters, our results reveal that the position of the cell does not influence the model output. The chosen UQ approach probed a minimum distance of 4.5 µm between the cells and a maximum distance of 22.5 µm. Our results suggest that the distance between the two cells does not impact the computed dielectric properties. For a tissue similar to cartilage with a small volume fraction, this finding is expected to hold true. However, the result cannot be generalised to other tissues with a higher cell density.

The variations in the numerically estimated dielectric properties can be attributed to variations in the cell volume over a wide frequency range. The cell orientation, as expressed by the angle with respect to its original orientation, plays a minor role. It has an impact around the *β*-dispersion where the prediction interval is not significantly widened. In sum, the cell volume is the parameter that dominantly influences the dielectric properties. Again, the uncertainty of the conductivity is small (less than 1% of the mean value) because the cells have a small impact on the overall conductivity. However, the permittivity changes drastically and the prediction interval includes a 25% variation of the mean value in the sub-MHz range. At higher frequencies, the permittivity is not impacted by the cell geometry.

**FIGURE 10:**
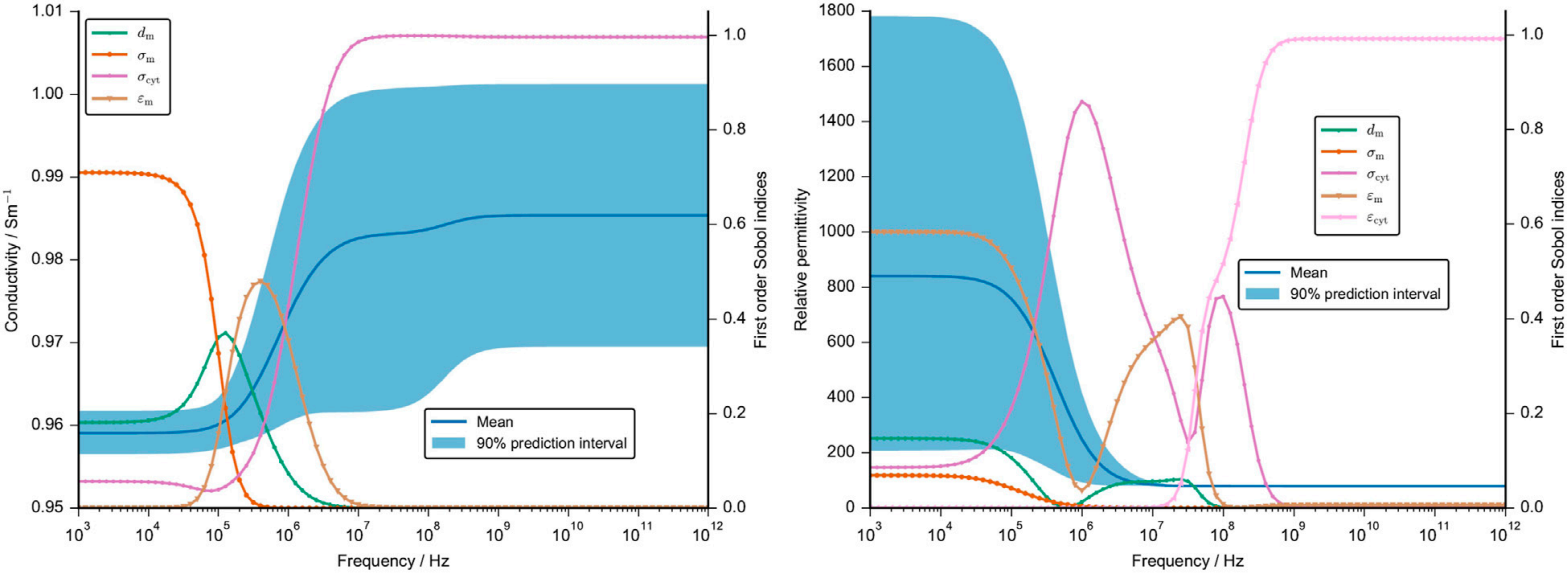
Mean value and 90% prediction interval of the conductivity (left) and the permittivity (right) are demonstrated over a frequency range. The frequency-dependent first order Sobol indices are presented for parameters with a Sobol index of more than 0.05. We neglected the uncertainty in the dielectric properties of the ECM and used fixed values. The assumptions for the UQ analysis of the other cellular dielectric parameter and the membrane thickness are given in Supplementary Table S3. Note that the cell volume, angle and position were set at a constant.

The second UQ analysis aimed at understanding the influence of the membrane thickness and the cellular dielectric properties on the numerical result. An initial analysis showed that the conductivity of the cartilaginous tissue strongly depends on the changes in the extracellular conductivity (refer to Supplementary Figure S13, S14). The permittivity is, however, also influenced by other parameters. To understand the influence of the cellular parameters in greater detail, we fixed the dielectric properties of the extracellular medium that overshadowed the contribution of the other parameters. This approach is supported by the fact that the extracellular properties could in principle be inferred from measurements of decellularised tissue with high accuracy, which would reduce their uncertainty drastically. The UQ analysis without the extracellular parameters shows a narrow 90% prediction interval of the computed conductivity before it widens from the start of the *β*-dispersion at about 1 MHz (Figure 10). At high frequencies, it roughly ranges from 0.96 S m^−1^ to 1 S m^−1^, which is again a very small deviation from the mean value. On the other hand, the 90% prediction interval of the estimated relative permittivity ranges from about 200 to 1,800 before the *β*-dispersion narrows significantly at high frequencies. This was to be expected as there was also no deviation between the different cartilage samples (Figure 7).

The frequency-dependent first-order Sobol indices reveal that up to 100 kHz, the cartilage conductivity depends on the membrane conductivity. The corresponding change in the impedance, however, is very small so it is questionable if this change could be measured (see Supplementary Figure S15). Around the *β*-dispersion, the membrane thickness and the membrane permittivity have the largest influence. At higher frequencies above 10 MHz, the cytoplasm conductivity is the sole parameter with a significant influence on the tissue conductivity. The tissue permittivity is primarily influenced by the membrane permittivity at low frequencies and by the cytoplasm permittivity around the *β*-dispersion (Figure 10). While the cytoplasm permittivity exhibits a large Sobol index at high frequencies, the corresponding uncertainty of the permittivity is negligibly small.

**FIGURE 11:**
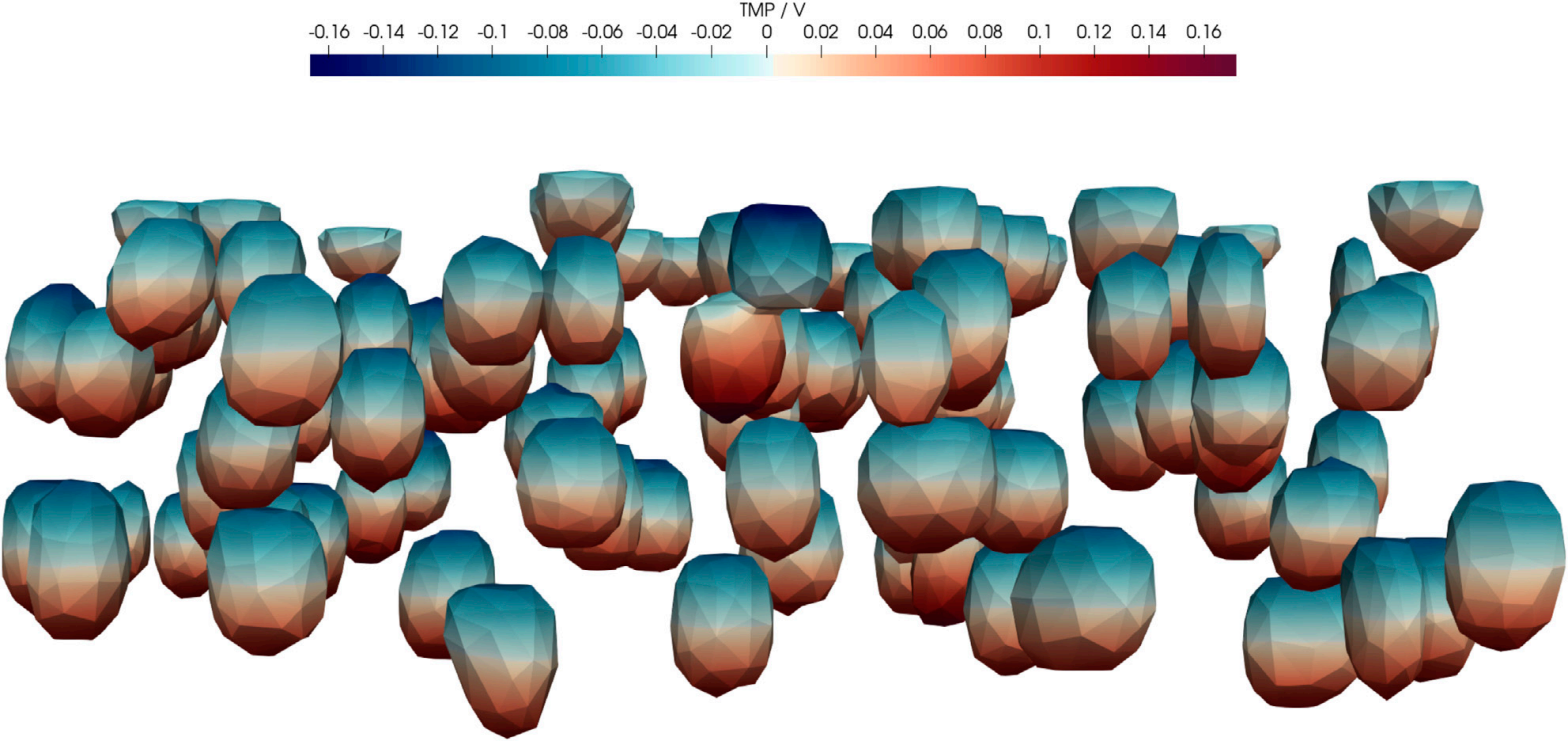
Real part of transmembrane potential (TMP) at 1 kHz for the average configuration of UQ (Case 2) given in Supplementary Table S3. In particular, *d*_m_ = 7 nm, *σ*_m_ = 0.8 μS m^−1^, *σ*_buf_ = 1 S m^−1^, *σ*_cyt_ = 0.48 S m^−1^, $\varepsilon_{r}^{\text{m}}=5.8$ , $\varepsilon_{r}^{\text{buf}}=80$ , $\varepsilon_{r}^{\text{cyt}}=60$ . The geometry was generated from the deep learning prediction of A4S1, and the applied voltage drop was 1 V.

An aspect that we have not covered in detail is the induced transmembrane potential. It may be used as an indicator for an effect of the electrical stimulation but its predictive quality is not entirely clear (Zimmermann et al., 2022a). Here, we computed the transmembrane potential for the average parameters of the UQ configuration (Figure 11).

## 4 Discussion

Osteoarthritis is a growing challenge in an ageing society. Cartilage tissue engineering is pursued as a possible cure but a viable treatment recipe is still elusive (Huey et al., 2012). In this work, we focused on the development of numerical models covering the interaction of electric fields and cartilage. Electric fields can be used in two ways with regard to cartilage tissue engineering: monitoring of the tissue state by assessing the dielectric properties and electrical stimulation to foster, for example, chondrogenesis.

The developed automated numerical workflow takes fluorescent microscopic images as an input and derives detailed tissue-specific 3D geometries. The geometries can be used in FEM simulations to estimate the dielectric properties of cartilage and also the local electric field. This is an important step forward to understand the interaction of electric fields and cartilage. It is in particular promising that the algorithm yields results for state-of-the-art fluorescence microscopy images within minutes if proper computational resources are available.

The first critical step of automated model generation is image segmentation. First, we established a batch processing of the 3D images using an established open-source software. This improves on previous studies that used custom Matlab scripts (Bennetts et al., 2014). The presented approach performs a number of pre-processing and cleaning steps before segmenting the image. Ellipsoids are then fitted to the segmented images. While this routine can be performed on a standard workstation, its automation is difficult due to noise or other imaging artefacts. Furthermore, the original approach proposed by Bennetts et al. (2014) has not covered the solution of meshing issues caused by intersecting cells. We found that intersecting cells pose a severe limitation for the algorithm as the combination of multiple ellipsoids can lead to irregular, ill-conditioned shapes.

Thus, machine learning and artificial intelligence-based solutions were tested to avoid the time-consuming pre-processing steps and directly segment the image. Initially, traditional machine learning algorithms were tested. It turned out that the performance of the classifier on noisy images after training is inadequate. Thus, traditional machine learning did not offer substantial benefits over the classical approach. A fine tuning of traditional machine learning could in principle be possible but is time-consuming and possibly computationally expensive.

Secondly, we investigated pre-trained deep learning models, i.e., models that were trained on imaging data of other cells and different dyes, imaging routines, etc. With this approach, we experienced a problem also referred to as ‘domain shift’ or ‘dataset shift’ problem (Uhlmann et al., 2022). We observed an underperformance and the generation of artefacts of the pre-trained models as they had to deal with new, unseen data. We attribute the encountered problem to a lack of pre-trained models for fluorescence microscopy 3D images in the public domain. To the best of our knowledge, accessible, high-quality fluorescence microscopy images are not widely available, and thus the pre-trained models could not be utilised to segment our images.

Hence, we switched to training from scratch using pre-defined model architecture and transfer learning, i.e., we trained an existing model with our own data. We chose deep learning algorithms because they can achieve state-of-the-art performance in 3D cell segmentation tasks and often outperform conventional machine learning algorithms (Caicedo et al., 2019; Prakash et al., 2020). Most importantly, we used original, unprocessed images directly as inputs, which eliminates the need for pre-processing as required by the classical approach. Our observations indicate that learning from scratch slightly outperforms transfer learning because using all pre-trained weights as starting points might lead to inappropriate or suboptimal initialisation of weights. Another way to improve the performance of transfer learning is to freeze the initial layers of the pre-trained model and leave only the final layers to be replaced and trained for the target task. Unfortunately, the *StarDist* implementation lacks this functionality at present. We found that our problem is solved sufficiently well if images segmented by the classical approach are used as the ground truth. In contrast to manually segmented images, they feature a well-defined ellipsoidal cell shape. This choice of the ground truth supports training in *StarDist*, which is a software specialised in blob-like cell shapes and features a minimal number of user-chosen parameters. As a result, even noisy images could be reliably segmented. However, the total number of cells was lower than those obtained from the classical method, resulting in a lower average cell volume. This affected the predicted impedance and dielectric properties. As the manual segmentation is biased because it relies on the expertise of the human who segments the image, the combination of impedance spectroscopy and fluorescence imaging together with full sample models can lead to a better reliability of the segmented images. During the acquisition of z-stacks in 3D fluorescence microscopy, the images can be stretched in *z*-direction due to a mismatch in the refractive index between the immersion medium and the samples (Diel et al., 2020). The uncorrected elongation along the *z*-axis can have an impact on the accurate estimation of the cell volume. Consequently, this can impact the FEM solutions. Our imaging data was not corrected and we can thus unfortunately not quantify the related uncertainty. In future research, the z-correction can be accomplished by using fluorescent beads with known geometries to mitigate volumetric measurement errors and minimize the loss of axial resolution. To address this issue, a practical tool in ImageJ, as proposed by Diel et al. (2020), can be employed.

In general, the segmentation by the trained deep learning model is significantly faster than the classical segmentation approach involving multiple processing steps. A drawback of deep learning segmentation is the computational requirements. A powerful GPU and a relatively large amount of memory are required to perform the training. The training itself also takes a considerable amount of time. On the other hand, the trained model can then be shipped and performs its task in a fraction of the required training time.

In a nutshell, we presented two competing approaches that can be successfully used to segment 3D fluorescence microscopy images of cartilage reliably in an automated manner. Both approaches were benchmarked against the average cell volume obtained by manual segmentation and performed well. If only a small number of images is available, researchers should use the classical segmentation approach as it can run on standard workstations and the increased processing time should not pose a problem. As soon as more images are available, the deep learning approach should be chosen. For that, a more powerful computer with a cutting-edge GPU should be used. About 20 images that are segmented by the classical approach are sufficient to train *StarDist*. Alternatively, our trained model could be tried but could suffer from ‘domain shift’ issues. Given the new data, our trained model can potentially serve as a valuable starting point to significantly reduce the training time. The average cell volume and its changes can already serve to detect joint inflammation or the initiation of osteoarthritis (Dore et al., 2010; Lv et al., 2019; Rim et al., 2020).

In this work, we are also concerned with an advanced biophysical model of the interaction between electric fields and cartilage. To build such a model, it is required to convert the image into a CAD geometry that is subsequently meshed. The deep learning-based workflow offers several benefits because the fast segmentation already leads to a surface mesh. The produced surface mesh was usually of high quality or could be fast and reliably optimised. In contrast, the classical approach required time-consuming post-processing of the CAD geometry and sometimes led to inferior mesh quality, which hampers automation. Moreover, a low mesh quality negatively impacts FEM solvers and leads to slower numerical convergence. Currently, another challenge is the cells that intersect with the image boundary due to a limited spatial resolution of the imaging approach. A possible alternative could be images obtained with tomography approaches that are capable of capturing entire cartilage samples (Wieland et al., 2021). Furthermore, the STL geometry creation step in the new approach allows for directly generating cell geometry of any shape from segmented images, eliminating the need for ellipsoid approximation. In order to convert STL geometry into CAD models, it is necessary to simplify and smooth the STL geometry. Despite thorough exploration of various algorithms, it is inevitable that this process will lead to a reduction in both the volume and surface area of the cell in the final CAD models. Smoothing the stair-like structure during surface reconstruction reduced cell surface area by approximately 15% while preserving cell volume within an acceptable biological error (about 5%). In future research, it could be attempted to use the STL geometries directly in *Netgen*. This approach would permit higher-order (i.e., curved) surface meshes.

We used the dielectric properties of the cartilage samples to compare both segmentation approaches. We could not find significant differences between the two approaches. Nevertheless, small deviations could be observed. We could correlate them to differences in the volume ratio of the geometries derived from the two segmentation approaches. A UQ analysis revealed that indeed the cell volume is the most influential parameter. In general, this finding implies two main conclusions. Firstly, an accurate segmentation of cellular images is required to reliably compare measured to predicted impedances. Secondly, it is most likely possible to estimate the cell volume from impedance measurements of cartilage samples. The latter is an important result because impedance measurements are significantly cheaper and faster than 3D imaging approaches and do not require dyes, i.e., are non-invasive. Thus, they could be used to monitor the *in-vivo* growth of artificial cartilage tissue.

A first step towards this goal is still the realisation of impedance spectroscopy experiments together with 3D fluorescence imaging. In this work, we used a simplistic parallel-plate capacitor geometry to apply the electric fields. In an experiment, such an electrode geometry would negatively impact the image acquisition. Instead, interdigitated electrodes integrated into cell culture wells could be used (Haas et al., 2010; Wolf et al., 2013). Thanks to the flexibility of the geometry preparation in *Netgen/NGSolve*, we expect no significant challenges in integrating different electrode geometries according to the experiment into our model. It will be the subject of future research to develop validated and calibrated electrode models combined with cell geometries derived from 3D images. Moreover, the approach will be extended to multicolour images to include not only the cell membrane. In principle, the cell volume could be scaled to include, for example, the pericellular matrix and nuclei in the final geometry. However, intersecting and touching cells found in real samples prevent in our experiences such an approach without manual interaction.

In addition to impedance spectroscopy, electrical stimulation for cartilage repair and regeneration can be studied with our approach. In future research, the numerical models of electrical stimulation can be coupled to impedance measurements to enable a so-called ‘digital twin’ (Zimmermann et al., 2021). The then-available accurate description of the electric fields the cells are exposed to can be expected to explain, which electrical stimulation protocol should be used as currently various waveforms, frequencies and amplitudes are considered (Vaca-González et al., 2017; Krueger et al., 2021; Zhou et al., 2023). Additionally, more data to improve the presented workflow will be generated. As the field of automated image segmentation is moving fast, we expect a growing number of data sets and improved software. Ultimately, the progress in the field will lead to more effective and reliable deep learning models for biomedical applications.

## 5 Conclusion

Overall, our study provides valuable insights into the development of an automated FEM mesh generation process from 3D cell images. The advantages and disadvantages of using artificial intelligence for volumetric image segmentation from fluorescence microscopy were emphasised. Employing deep learning-based mesh generation allows for high-quality meshes without the requirement for CAD geometry post-processing and reduces overall computational time. The findings of this study are significant for researchers and engineers working on large-scale FEM simulations based on 3D images of cell structures and biological tissues. With further optimisation and an increased data size, it can be expected that almost instant processing (i.e., segmentation and numerical analysis) of the imaging data can become feasible. This can pave the way for sample-specific interventions to foster, for example, cartilage tissue engineering approaches.

## Data Availability

The datasets presented in this study can be found in online repositories. The names of the repository/repositories and accession number(s) can be found below: Zenodo, URL: dx. doi.org/10.5281/zenodo.7930409.

## Footnotes

1
https://imagej.net/software/fiji/

2
https://dev.opencascade.org/

3
https://github.com/trasse/model_zoo

4
https://scikit-image.org/

5
https://www.freecadweb.org/

6
https://github.com/j-zimmermann/uncertainpy

7
https://networkx.org/
