# Supplementary material for: Contributions of deep learning to automated numerical modelling of the interaction of electric fields and cartilage tissue based on 3D images
Source: Front Bioeng Biotechnol. 2023 Aug 29;11:1225495. doi: 10.3389/fbioe.2023.1225495 (PMC10497969; doi:10.3389/fbioe.2023.1225495)
Supplement: Supplementary file 1 [file DataSheet1.pdf]

Supplementary Material

1 SUPPLEMENTARY DATA

1.1 The procedure of manual cell volume estimation from Lv et al. (2019)

To estimate the cell volume, Bitplane Imaris v7 (trial version) [1] was used for cell segmentation.

- A Gaussian filter was applied to a region of interest (ROI).
- A filter width was selected based on voxel sizes.
- A sphere diameter was specified based on the minimum expected object diameter in the dataset to perform background subtraction.
- If background subtraction is not selected, a absolute intensity threshold is applied.
- A cell volume was calculated after filling the holes and removing manually the outliers, such as small or large objects.

1.2 Machine learning-based image segmentation

1.2.1 Trainable Weka Segmentation

Initially, we tried a trainable machine learning approach. We used the Trainable Weka Segmentation (TWS) method (Arganda-Carreras et al., 2017), which is well-established for microscopy pixel classification and available in the popular software *ImageJ*. First of all, because only one image can be loaded when training data, we chose ten slices from four images (two good-quality and two noisy images) to create one single image. This approach aimed to encompass a wide range of pixel intensities present in our dataset. A set of training pixels (STP) for each class (cell and background) were manually annotated on different slices by drawing traces using the Graphical User Interface (GUI). Although an acceptable classification with a small number of annotations can be obtained (Arganda-Carreras et al., 2017), we have marked 80 STPs for each class. In particular, the image has 80 STPs marked at different slices of 3D images using the cursor on the interactive GUI. In the setting panel, training features were kept as default in TWS, and image segmentation is obtained employing the Fast Random Forest algorithm (Breiman, 2001). The training was conducted on a workstation with Intel Core i7-10700 (2.90 GHz, 16 MB) and 64 GB of RAM.

The TWS training time is roughly 5 hours. The TWS classifier demonstrated excellent performance with high-quality images. However, for noisy images, the model produced relatively unsatisfactory results (as shown for example in Figure S1). Further, the TWS cannot separate touching objects or connect different parts belonging to one cell due to uneven distribution of dye, despite several attempts to manually mark the ground truth as precisely as possible. Consequently, the *Distance Transform Watershed* is required to apply once the image is fully segmented. Due to the satisfactory results achieved by the classical approach, we did not further fine-tune the TWS classifier.

1.2.2 *DeepImageJ*

An alternative method is to segment 3D fluorescent images using deep learning. Many software libraries and applications have surfaced in recent years. After an initial assessment, we focused on a selection of software solutions that in our opinion covered the field well enough. We assessed pre-trained deep learning

[1] `https://imaris.oxinst.com/`

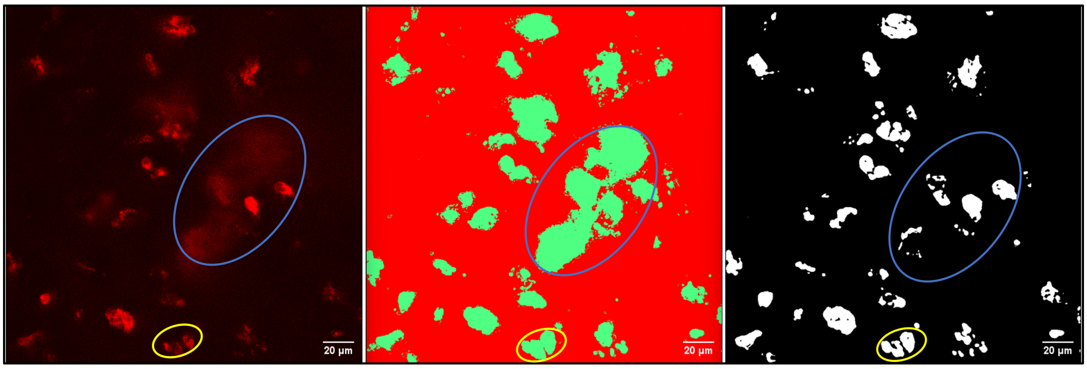

Figure S1. 2D view of the middle slice from a noisy image. From left to right: the original image, the result of the TWS segmentation and the result of the classical segmentation after applying auto-threshold (the thresholding image), respectively. The TWS model performed poorly on noisy images (indicated by the blue ellipse) and was unable to separate touching objects or connect different parts of a cell (marked by the green ellipse), despite manual ground truth marking. As a result, the use of *Distance Transform Watershed* is necessary for post-segmentation processing.

models from *DeepImageJ* (Gómez-de Mariscal et al., 2021) because they are available in *ImageJ*, which we already used for the classical approach.

We searched for 3D pre-trained models online[2]. It turned out that most of the available models are either for electron microscopy (EM) or for detecting cell boundaries. We found only two models that were used for cell segmentation from confocal microscopy (Wolny et al., 2020; Bondarenko et al., 2022). The aforementioned deep learning models utilised a 3D U-Net (Ronneberger et al., 2015). Additionally, the three most popularly downloaded EM models were tested. However, neither of these models could be successfully applied to our data. We believe that the main reason for the failure is due to the differences between the training data and our data. The images used for pre-training were from different cell types in different tissues (such as cartilage, plants, and mouse embryos) and were acquired using different dyes. Thus, the training images may have had a different contrast, noise, and background intensity compared to our data.

1.2.3 Cloud-based Deep Learning Platforms

As the pre-trained models did not turn out to be capable of segmenting our data, we decided to train deep learning models on our specific dataset.

Biomedisa (Lösel et al., 2020) and *ZeroCostDL4Mic* (von Chamier et al., 2021) were chosen because they are examples of cloud-based solutions and do not require own hardware. *Biomedisa* supports the training of CNN for the segmentation of 3D image data based on a 3D U-Net and is accessible through a web browser connected to a computing cluster. *ZeroCostDL4Mic* is a platform hosted on Google Colab and offers multiple segmentation algorithms, including also *StarDist*. A free version of Google Colab offers two Intel Xeon CPUs with one core and 12 GB RAM, and an NVIDIA Tesla K80 GPU with 12 GB VRAM and a runtime of up to 12 hours. A step-by-step instruction was outlined in the self-explanatory Jupyter Notebooks within the *ZeroCostDL4Mic* and in *Biomedisa*'s website[3], eliminating the need for extensive computational expertise.

[2] `https://bioimage.io/\#/?type=all&tags=deepimagej,3d`

[3] `https://biomedisa.org/`

However, in both cases, we experienced out-of-memory errors indicating that the provided cloud hardware was not sufficient to train the provided algorithms with our data.

1.2.4 *CellPose3D*

Cellpose3D is an extension of the *Cellpose* algorithm (Stringer et al., 2020) designed for 2D application. It utilises the full 3D information considering the spatial context and volumetric characteristics of cells to enhance the segmentation accuracy on 3D image data. It also offers simplified training data preparation and instance reconstruction compared to the original version. We also investigated transfer learning on a pre-trained *Cellpose3D model*. The model was initially trained on 125 3D stacks of fluorescent confocal microscopy images depicting cell membranes in the meristem of the plant model organism A. thaliana. We fine-tuned the model with our dataset. The *CellPose3D* model underwent training and testing using identical training and testing data as the *StarDist* model. The data was prepared according to the instructions[4] to meet the requirements of the *CellPose3D* model, which includes formatting and storing the data in an HDF5 file format. The default configuration settings were maintained throughout the training process, conducted on the post-processing node of the HAUMEA HPC cluster. The *CellPose3D* involves two primary steps in prediction. The network is first used to generate predictions for gradient fields and foreground segmentation based on the image data. Post-processing techniques are then employed to refine network predictions and reconstruct instance segmentation. The original code, training pipelines, and application pipelines are openly accessible at `https://github.com/stegmaierj/Cellpose3D`. Our modified scripts regarding to our specific application is given at `dx.doi.org/10.5281/zenodo.7930409`.

The *CellPose3D* models were trained for approximately 20 hours until the loss values reached convergence after 300 epochs. In all predicted images, no cells could be detected (see Figure S2). The failure of the predictions was due to the foreground-predicted images, as they identified almost the entire area as the foreground. Given the considerable increase in training and prediction times compared to *StarDist* as well as the additional data preparation step, we did not pursue further investigation into the algorithm failure.

1.2.5 *ilastik*

ilastik offers machine-learning-based bioimage analysis with a user-friendly interface. Its interactive workflow does not require a large 3D training dataset. Machine learning models for cell segmentation can be trained efficiently and intuitively in *ilastik* without extensive coding experience. The *ilastik* segmentation pipeline involved two main steps using its interactive GUI[5]. First, we performed pixel classification by training a random forest classifier to distinguish between background and cytoplasm. We manually annotated voxels until additional training data no longer improved segmentation results on the training data. Training features were optimised using automatic feature suggestions provided by *ilastik*. Second, we identified individual cells by applying the hysteresis thresholding method to the results of the previous step. This step is an unsupervised technique that does not require any training data. Following the examination of various threshold value combinations, we determined that a threshold of 0.85 and 0.4 provided the most appropriate results for our specific case. The high threshold (core) and low threshold (final) are employed to differentiate and segment touching objects for detection purposes. The main paper includes a description of the results, providing a comparison with the *StarDist* algorithm.

[4] `https://github.com/stegmaierj/Cellpose3D`

[5] `https://www.ilastik.org/documentation/objects/objects`

Table S1. The effect of learning rate and the U-Net depth on the validation accuracy at the IoU threshold of 0.5. N.A indicates that a prediction process failed to due to an out of memory.

U-Net depth		Learning rate 0.001	0.0003	0.0001	0.00005
1	Validation accuracy	0.47	0.51	0.55	0.47
	Training accuracy	0.58	0.65	0.62	0.58
	Training time (hour)	4.61	4.65	4.68	4.69
2	Validation accuracy	0.55	0.59	0.60	0.58
	Training accuracy	0.65	0.70	0.67	0.65
	Training time (hour)	4.73	4.81	4.82	4.83
3	Validation accuracy	N.A	0.50	0.47	0.52
	Training accuracy	N.A	0.59	0.63	0.62
	Training time (hour)	4.81	4.84	4.88	4.90

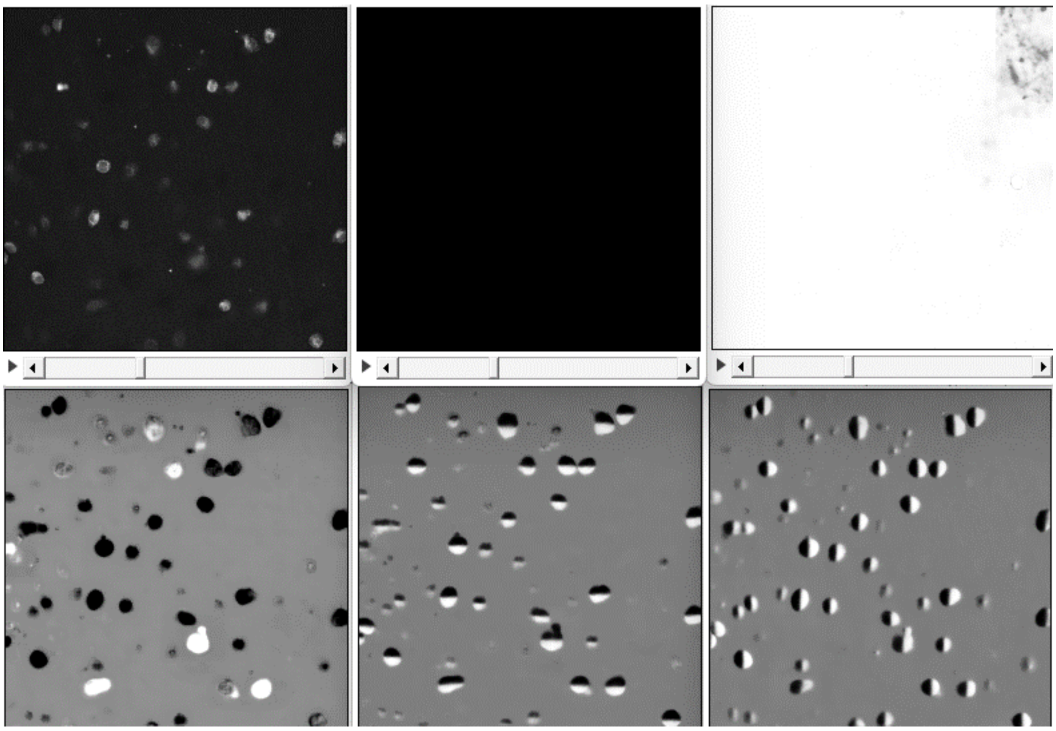

Figure S2. From top left to top right: the original image of A1S1, the final prediction from *CellPose3D*, and the foreground prediction from the neural network. From Bottom left to right: the neural network's predicted gradient maps in x,y and z-direction, respectively. The middle panel on the top row is the final predicted image reconstructed from the foreground prediction and the predicted gradient maps. No cells were detected in the final prediction. Its failure can be attributed to foreground identification, as a significant portion of the image area was erroneously classified as foreground (in white).

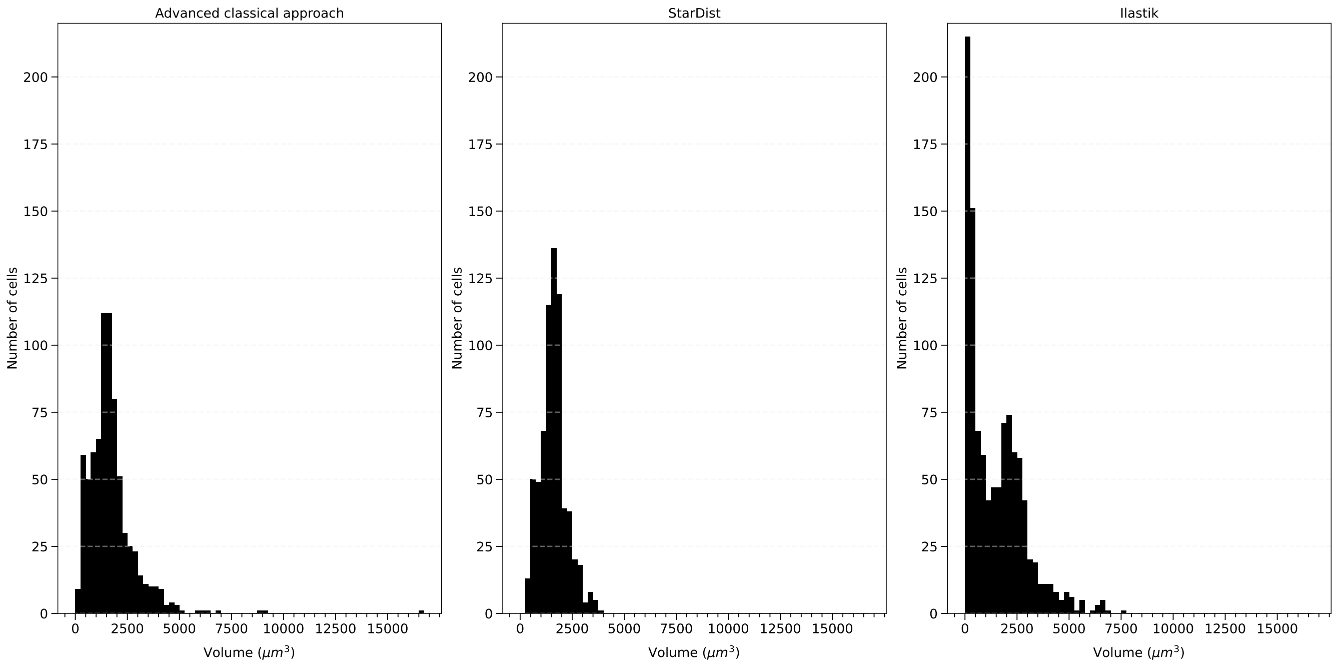

Figure S3. From top left to top right: the histogram of the cell volumes from the advanced classical segmentation, *StarDist* and *ilastik* approaches. Using the advanced classical approach, a few artifacts with volumes exceeding $5000\,\mathrm{\mu m}^3$ were misidentified as cells. These artifacts had similar pixel intensities to cells and couldn't be filtered based on volume alone. They were excluded from the final geometry using ellipsoid volume measurements. In contrast, *StarDist* produced reasonable cell volumes, all below $4000\,\mathrm{\mu m}^3$. This approach avoids predictions of objects that deviate significantly in size from the training data. *ilastik* detected certain cell parts as separate objects, leading to an increased cell count and smaller average cell volume.

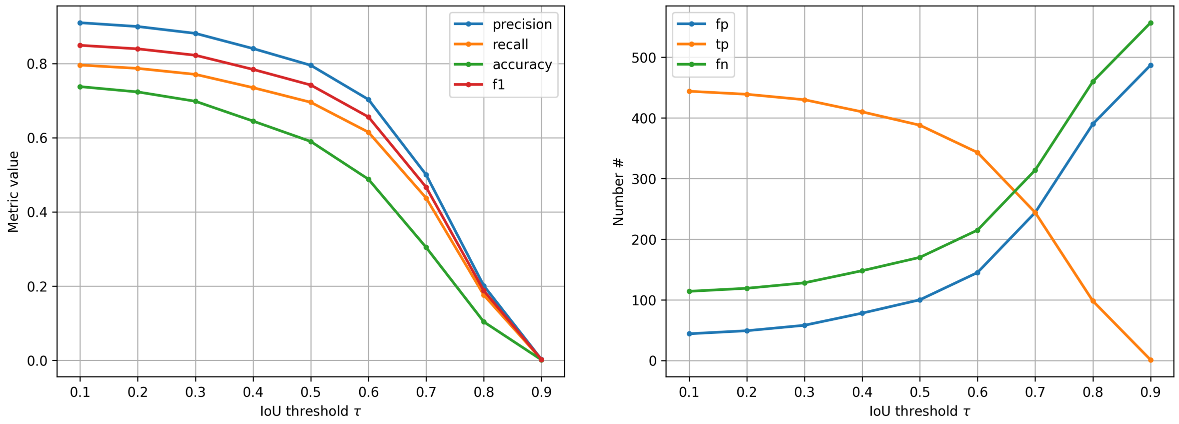

Figure S4. The $precision$, $recall$, $F1(Dice)$ score, $accurary$, true positive (TF), false positives (FP), and false negatives (FN) with different IoU threshold τ for the validation dataset.

Table S2. The $precision$, $recall$, $F1$ score and $accuracy$ with IoU threshold τ of 0.5 for validation and test dataset of the *StarDist* model.

Dataset	precision	recall	F1 score	accuracy
Validation	0.80	0.70	0.74	0.59
Test	0.88	0.90	0.89	0.80

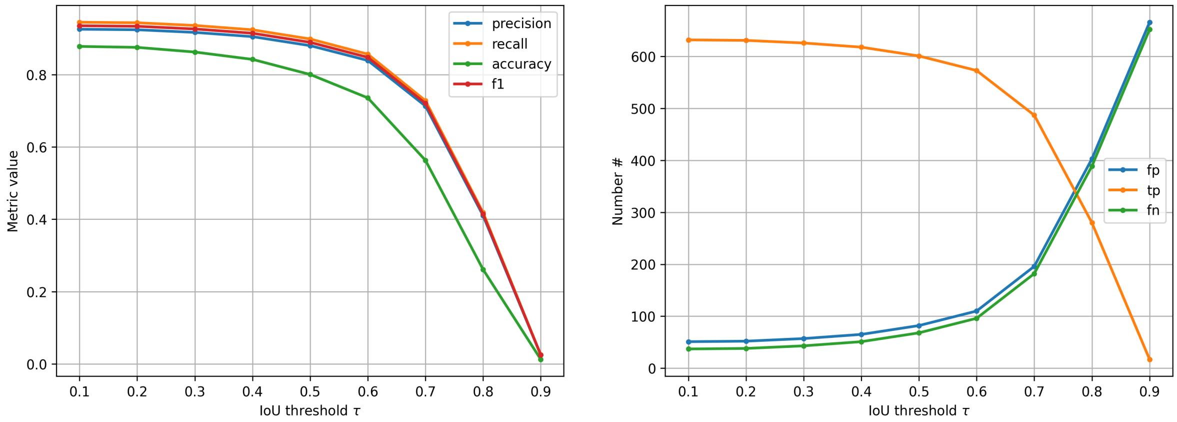

Figure S5. The $precision$, $recall$, $F1(Dice)$ score, $accurary$, true positive (TF), false positives (FP), and false negatives (FN) with different IoU threshold τ for the testing data.

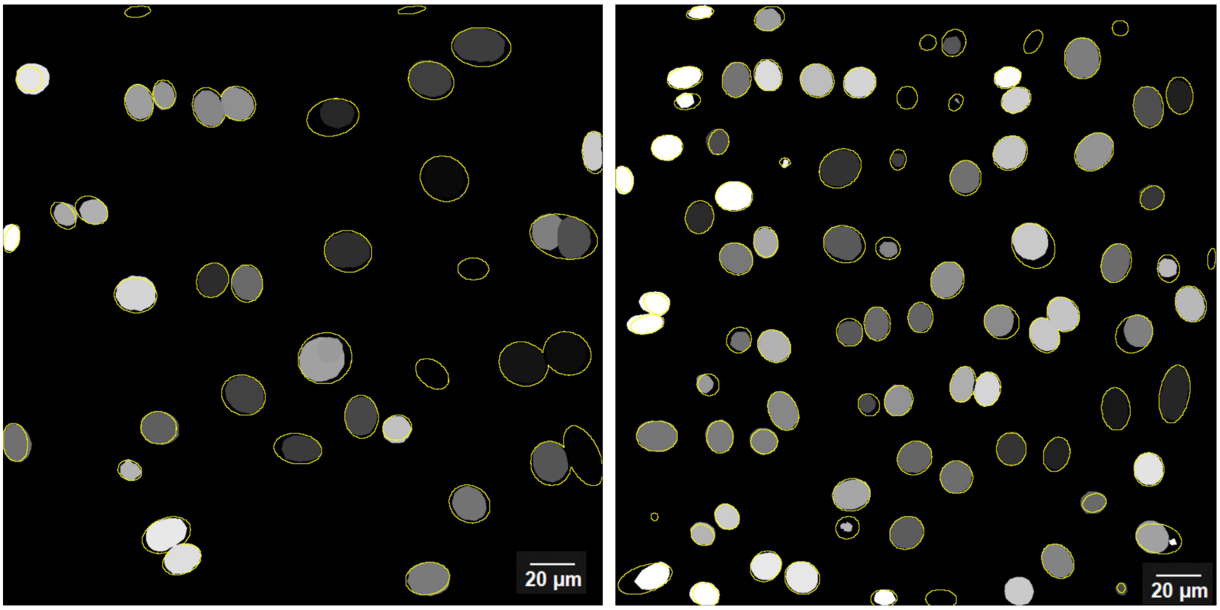

Figure S6. Superimposed positions of the expected output (the yellow ellipsoids) onto the deep learning created masks. The yellow ellipsoids highlight the regions that are expected to be segmented, while the deep learning created masks show the actual segmentation results. The majority of cells were detected, although some of their volumes were slightly smaller compared to the ground truth.

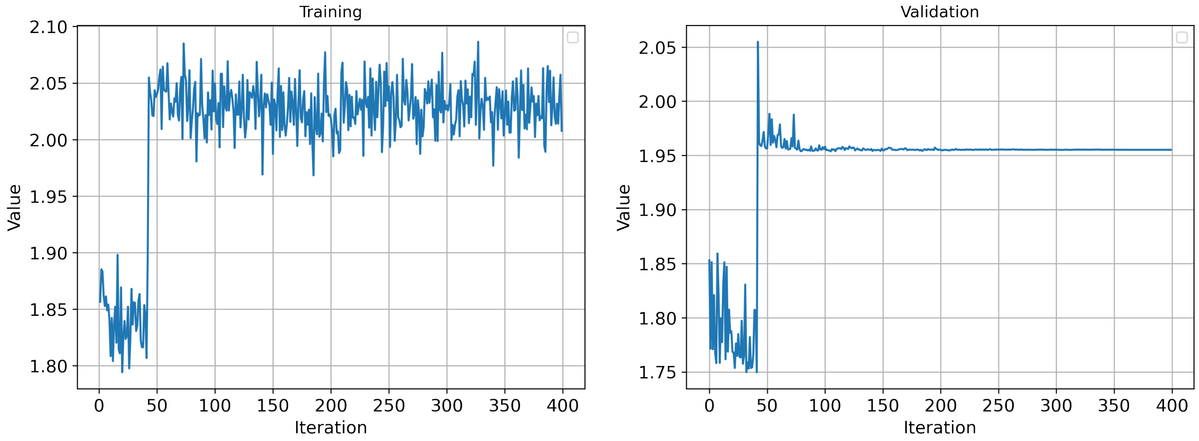

Figure S7. Training loss curve during the training and validation process with the learning rate of 0.1 and the U-Net depth of 2. The loss did not show any improvement as it increased after 50 epochs and remained relatively constant.

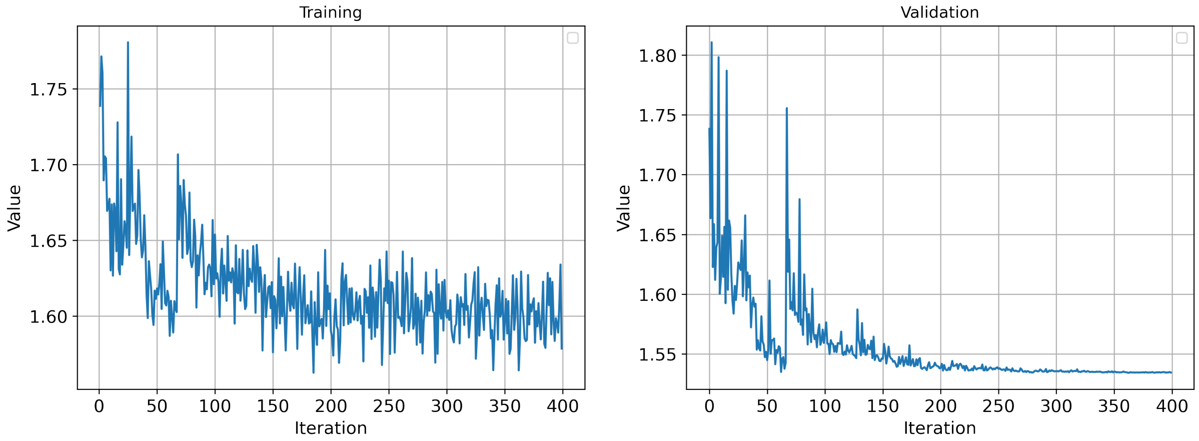

Figure S8. Training loss curve during the training and validation process with the learning rate of 0.01 and the U-Net depth of 2. The training loss is better than the case with the learning rate of 0.1, but still both training and validation loss did not exhibit any significantly improvement.

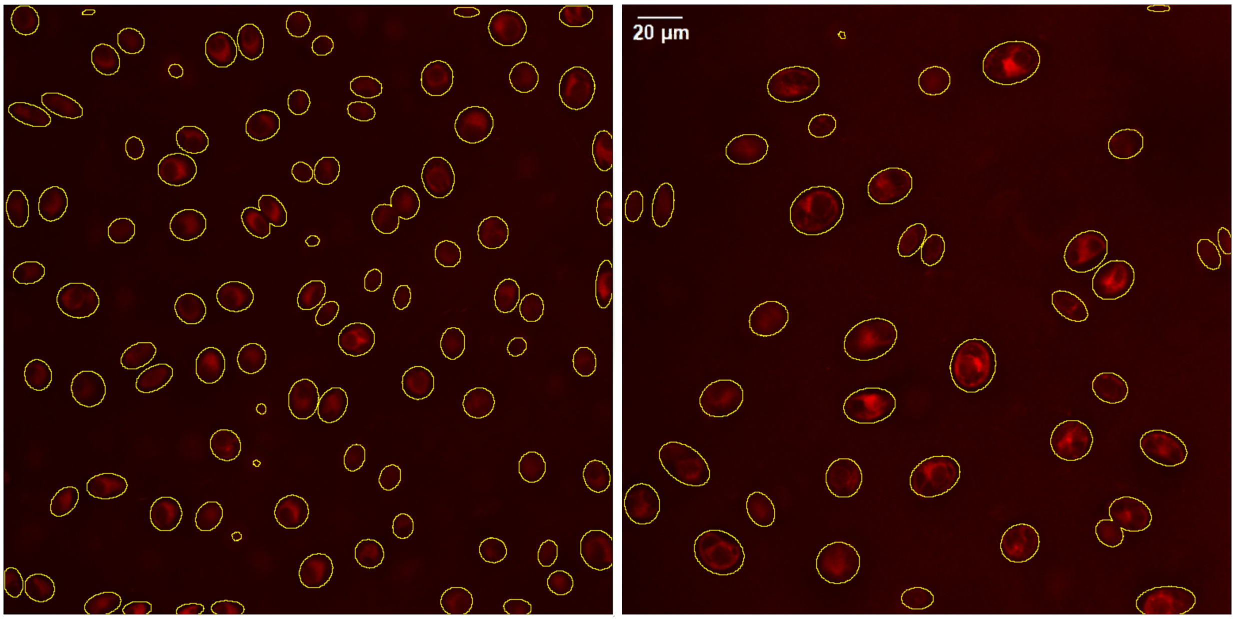

Figure S9. Overlay image of fitted ellipsoids and the original cells. The ellipsoids effectively represent cell structures.

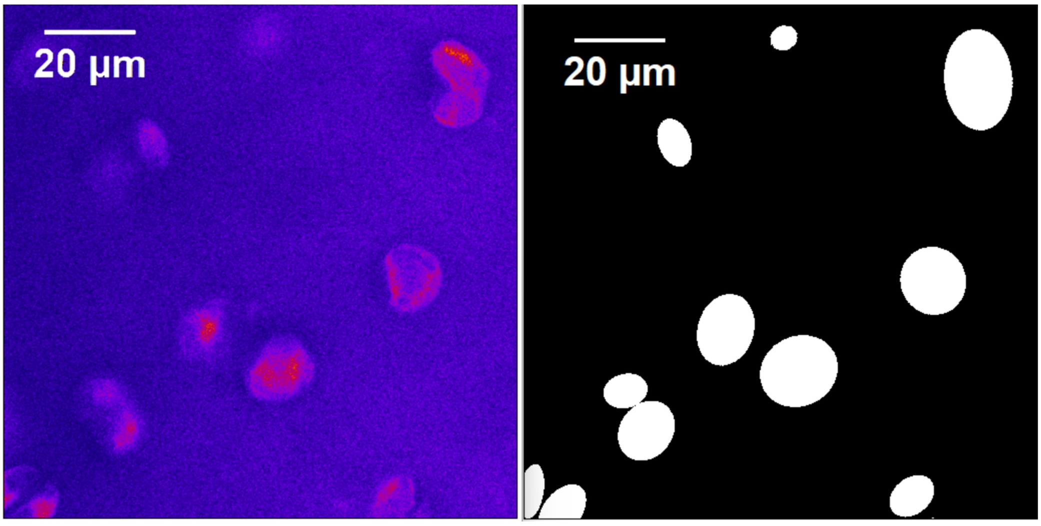

Figure S10. An example of a slice in a training pair. Left: Input data as an original image. For visualization purposes, the Look-Up Tables (LUT) in ImageJ were modified to the ”Fire” color scheme. Right: Ground true as a fitted ellipsoid image.

Table S3. Model parameters for UQ given as a function of the uniform distribution $\mathcal{U}$. x, y, z: coordinates of the cell, V_{scale}: scaling factor of the volume, θ: angle of the cell with respect to its local axis, d_{m}: membrane thickness, σ_{m}: conductivity of the cell membrane, σ_{buf}: conductivity of the cell culture medium, σ_{cyt}: conductivity of the cytoplasm, $\varepsilon_r^{\mathrm{m}}$: relative permittivity of the cell membrane, $\varepsilon_r^{\mathrm{buf}}$: relative permittivity of the cell culture medium, $\varepsilon_r^{\mathrm{cyt}}$: relative permittivity of the cytoplasm

Case	Parameter	Distribution	Reasoning
Case 1	x / µm	$\mathcal{U}(-10, 10)$	Assumptions for position error in x-axis
	y / µm	$\mathcal{U}(-10, 10)$	Assumptions for position error in y-axis
	z / µm	$\mathcal{U}(-10, 10)$	Assumptions for position error in z-axis
	V_{scale}	$\mathcal{U}(0.8, 1.2)$	Scaling factor of the cell volume
	θ / °	$\mathcal{U}(10, 90)$	Rotation angle to cover orientation error
Case 2	d_{m} / nm	$\mathcal{U}(3.5, 10.5)$	Assumptions from (Ermolina et al., 2000)
	σ_{m} / $\mu\mathrm{S\,m^{-1}}$	$\mathcal{U}(0.08, 56)$	Assumptions from (Ermolina et al., 2000)
	σ_{buf} / $\mathrm{S\,m^{-1}}$	$\mathcal{U}(0.95, 1.05)$	Assumptions from (Fear and Stuchly, 1998)
	σ_{cyt} / $\mathrm{S\,m^{-1}}$	$\mathcal{U}(0.033, 1.1)$	Assumptions from (Ermolina et al., 2000)
	$\varepsilon_r^{\mathrm{m}}$	$\mathcal{U}(1.4, 16.8)$	Assumptions from (Ermolina et al., 2000)
	$\varepsilon_r^{\mathrm{buf}}$	$\mathcal{U}(60, 80)$	Assumptions from (Escobar et al., 2020) and (Zimmermann et al., 2022)
	$\varepsilon_r^{\mathrm{cyt}}$	$\mathcal{U}(60, 77)$	Assumptions from (Ermolina et al., 2000)

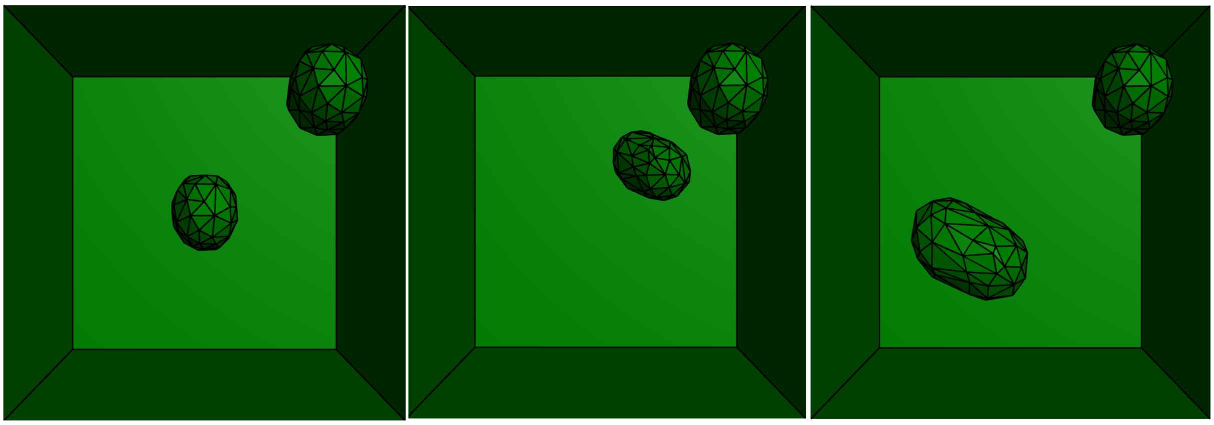

Figure S11. An illustration of geometries for UQ of the geometrical variation. One cell was always fixed at the top right corner of the box. A copy of the fixed cell was modified as shown in the three panels from left to right: the cell was placed in the centre of the square box; the cell was scaled to 80% of its volume, rotated by $45°$ and located at $x = y = z = 10\,\mu\mathrm{m}$; the cell volume was increased by 20%, rotated by $90°$ and located at $x = y = z = -10\,\mu\mathrm{m}$. The parameter combination in the above geometries was chosen for visualising examples of the geometrical models used in the UQ approach. The actual combination of cell location, rotation angle and volume scaling factor used in the UQ runs relies upon the algorithm and parameter distribution.

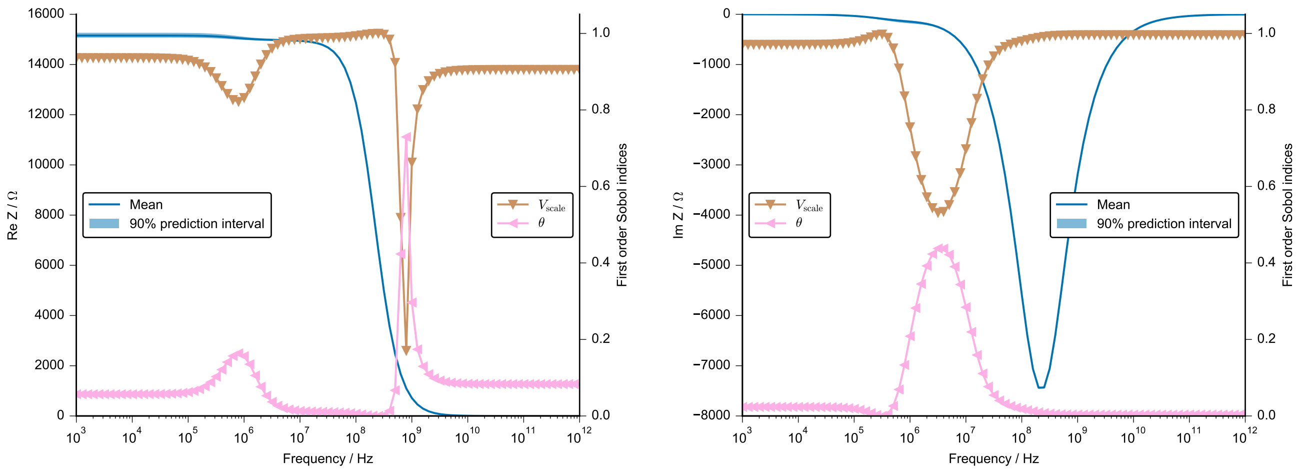

Figure S12. The mean value and 90% prediction interval of the real part (left) and the imaginary part (right) of the impedance are demonstrated over a frequency range. The frequency-dependent first order Sobol indices are presented for parameters with a Sobol index of more than 0.05. The assumptions for the UQ analysis of the geometrical parameter, namely x, y, z position, volume and cell angle, are given in Table 1. Note that the other cellular dielectric properties were set at a constant.

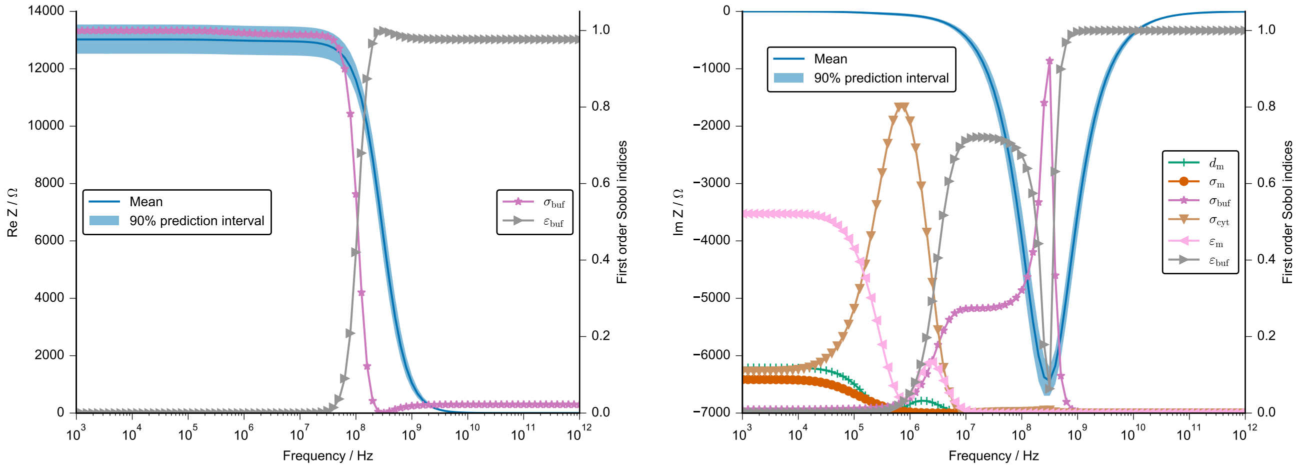

Figure S13. The mean value and 90% prediction interval of the real part (left) and the imaginary part (right) of the impedance are demonstrated over a frequency range. The frequency-dependent first order Sobol indices are presented for parameters with a Sobol index of more than 0.05. The assumptions for the UQ analysis of the cellular dielectric parameter and the membrane thickness are given in Table 1. Note that the cell volume, angle and position were set at a constant.

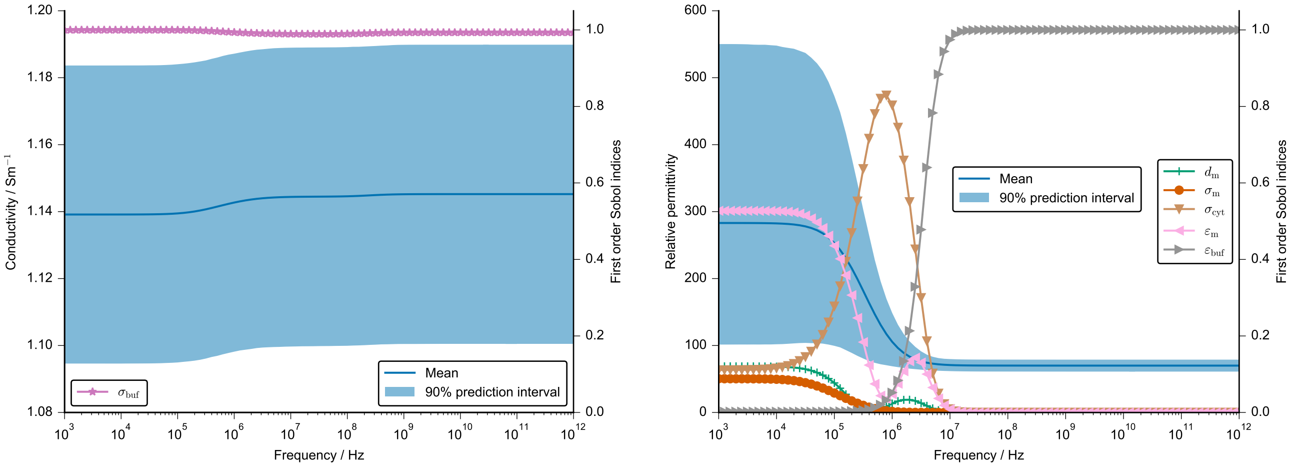

Figure S14. The mean value and 90% prediction interval of the conductivity (left) and the permittivity (right) are demonstrated over a frequency range. The frequency-dependent first order Sobol indices are presented for parameters with a Sobol index of more than 0.05. The assumptions for the UQ analysis of the cellular dielectric parameter and the membrane thickness are given in Table 1. Note that the cell volume, angle and position were set at a constant.

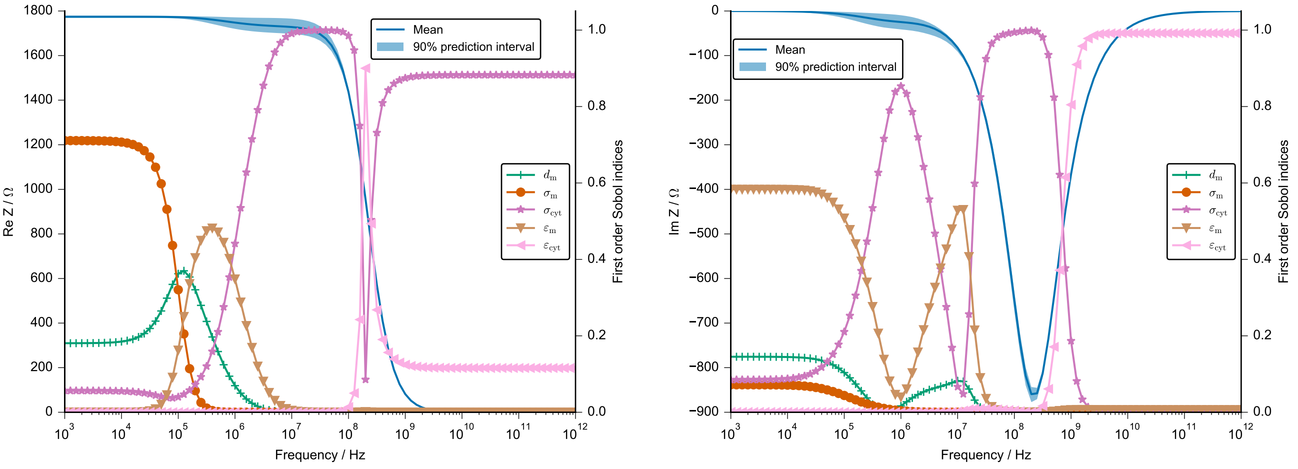

Figure S15. The mean value and 90% prediction interval of the real part (left) and the imaginary part (right) of the impedance are demonstrated over a frequency range. The frequency-dependent first order Sobol indices are presented for parameters with a Sobol index of more than 0.05. On the contrary to the results in Fig. S13, we neglected the uncertainty in the dielectric properties of the ECM and used fixed values. The assumptions for the UQ analysis of the cellular dielectric parameter and the membrane thickness are given in Table 1. Note that the cell volume, angle and position were set at a constant.

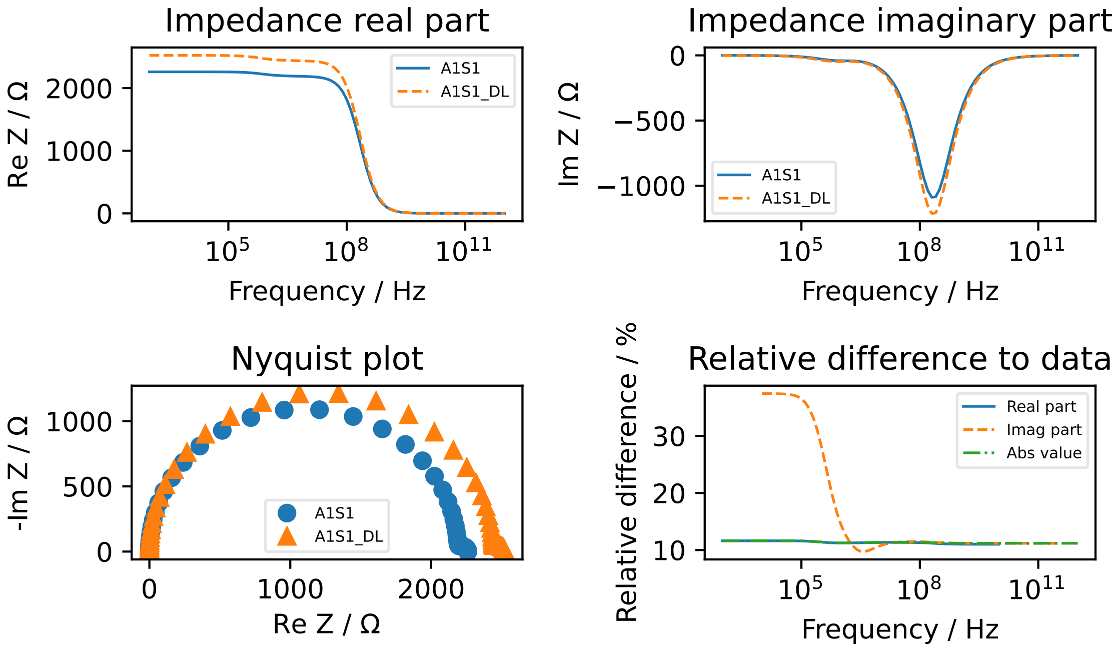

Figure S16. The impedance plot of the A1S1 sample using the deep learning approach (dashed line and denoted by DL) and the classical approach (straight line).

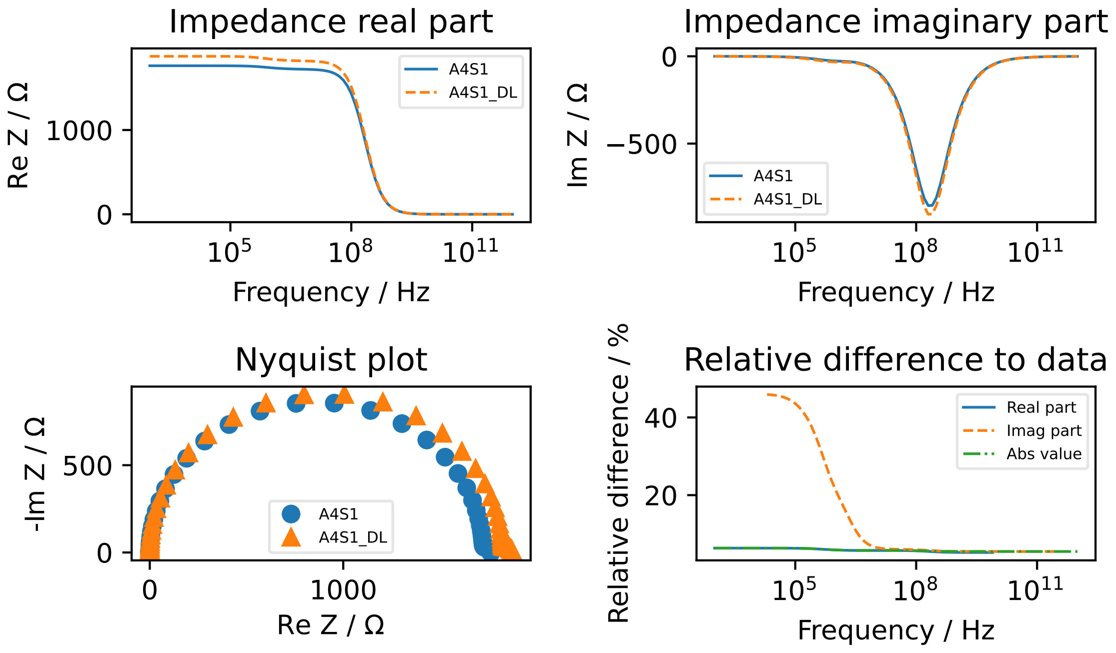

Figure S17. The impedance plot of the A4S1 sample using the deep learning approach (dashed line and denoted by DL) and the classical approach (straight line).

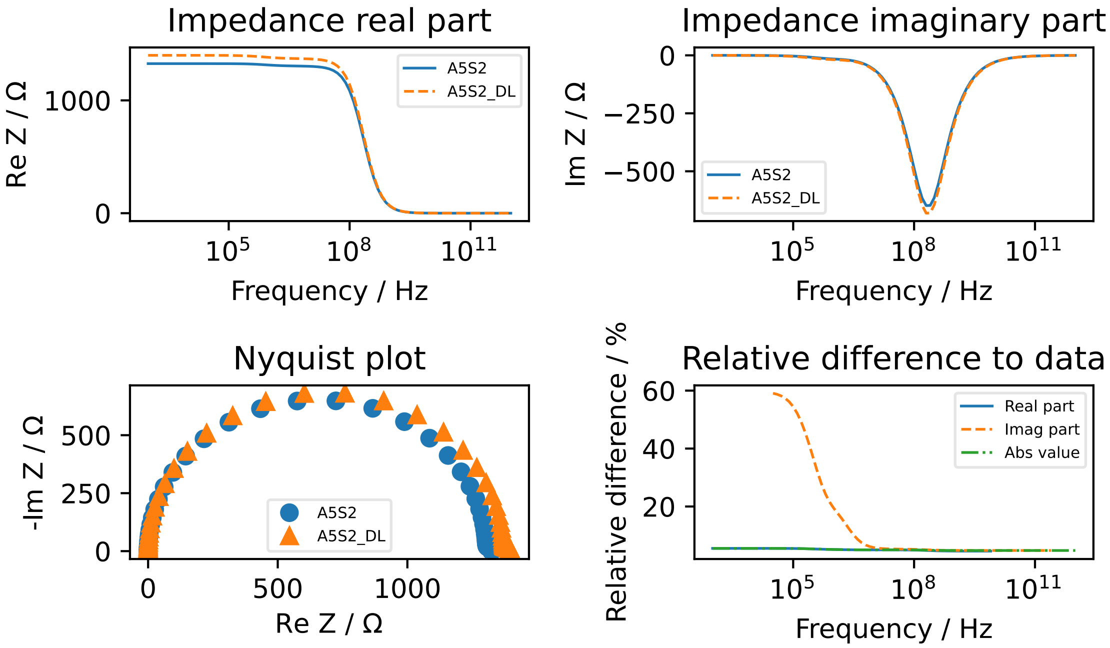

Figure S18. The impedance plot of the A5S2 sample using the deep learning approach (dashed line and denoted by DL) and the classical approach (straight line).

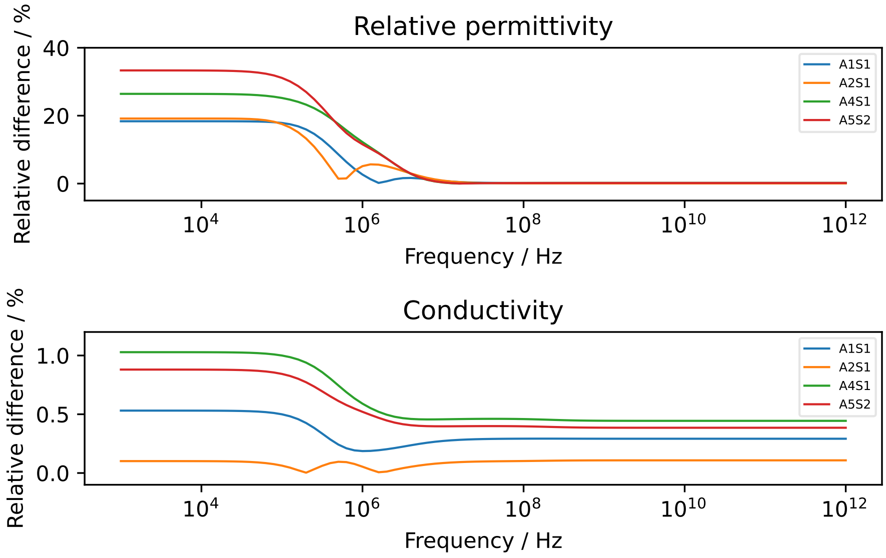

Figure S19. The relative difference of the conductivity and permittivity of an individual sample using classical and deep learning segmentation in the meshing workflow.

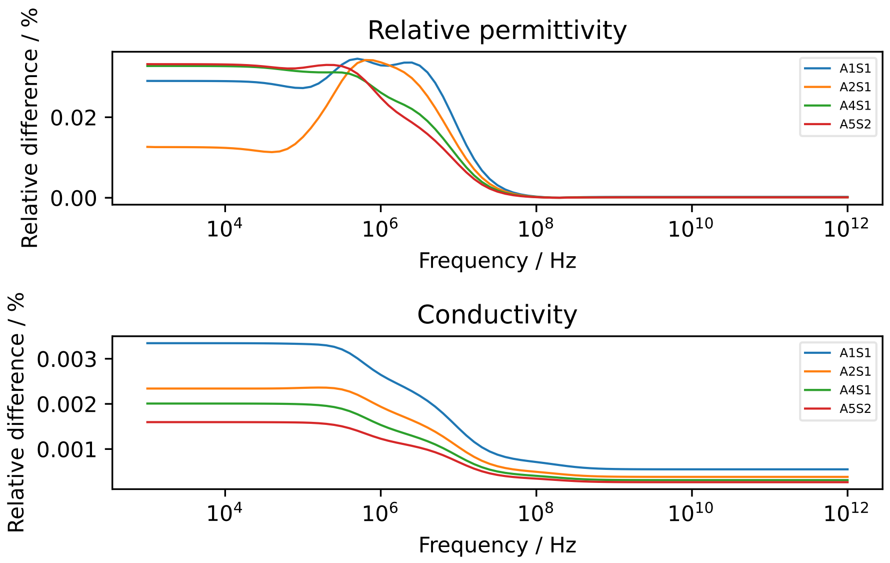

Figure S20. The relative difference of the computed dielectric properties using moderate and fine mesh size.

REFERENCES

Arganda-Carreras, I., Kaynig, V., Rueden, C., Eliceiri, K. W., Schindelin, J., Cardona, A., et al. (2017). Trainable weka segmentation: a machine learning tool for microscopy pixel classification. *Bioinformatics* 33, 2424–2426. doi:10.1093/bioinformatics/btx180

Bondarenko, V., Nikolaev, M., Kromm, D., Belousov, R., Wolny, A., Rezakhani, S., et al. (2022). Coordination between embryo growth and trophoblast migration upon implantation delineates mouse embryogenesis. *bioRxiv* , 2022–06doi:10.1101/2022.06.13.495767

Breiman, L. (2001). Random forests. *Machine Learning* 45, 5–32. doi:10.1023/a:1010933404324

Ermolina, I., Polevaya, Y., and Feldman, Y. (2000). Analysis of dielectric spectra of eukaryotic cells by computer modeling. *European Biophysics Journal* 29, 141–145. doi:10.1007/s002490050259

Escobar, J. F., Vaca-González, J. J., Guevara, J. M., and Garzón-Alvarado, D. A. (2020). Effect of magnetic and electric fields on plasma membrane of single cells: A computational approach. *Engineering Reports* 2. doi:10.1002/eng2.12125

Fear, E. and Stuchly, M. (1998). Modeling assemblies of biological cells exposed to electric fields. *IEEE Transactions on Biomedical Engineering* 45, 1259–1271. doi:10.1109/10.720204

Gómez-de Mariscal, E., García-López-de Haro, C., Ouyang, W., Donati, L., Lundberg, E., Unser, M., et al. (2021). DeepImageJ: A user-friendly environment to run deep learning models in ImageJ. *Nature Methods* 18, 1192–1195. doi:10.1101/799270

Lösel, P. D., van de Kamp, T., Jayme, A., Ershov, A., Faragó, T., Pichler, O., et al. (2020). Introducing biomedisa as an open-source online platform for biomedical image segmentation. *Nature Communications* 11. doi:10.1038/s41467-020-19303-w

Lv, M., Zhou, Y., Polson, S. W., Wan, L. Q., Wang, M., Han, L., et al. (2019). Identification of chondrocyte genes and signaling pathways in response to acute joint inflammation. *Scientific Reports* 9. doi:10.1038/s41598-018-36500-2

Ronneberger, O., Fischer, P., and Brox, T. (2015). U-Net: Convolutional networks for biomedical image segmentation. In *Lecture Notes in Computer Science* (Springer International Publishing), 234–241. doi:10.1007/978-3-319-24574-4_28

Stringer, C., Wang, T., Michaelos, M., and Pachitariu, M. (2020). Cellpose: a generalist algorithm for cellular segmentation. *Nature Methods* 18, 100–106. doi:10.1038/s41592-020-01018-x

von Chamier, L., Laine, R. F., Jukkala, J., Spahn, C., Krentzel, D., Nehme, E., et al. (2021). Democratising deep learning for microscopy with ZeroCostDL4mic. *Nature Communications* 12. doi:10.1038/s41467-021-22518-0

Wolny, A., Cerrone, L., Vijayan, A., Tofanelli, R., Barro, A. V., Louveaux, M., et al. (2020). Accurate and versatile 3d segmentation of plant tissues at cellular resolution. *Elife* 9, e57613. doi:10.1101/2020.01.17.910562

Zimmermann, J., Altenkirch, R., and van Rienen, U. (2022). Numerical study on the effect of capacitively coupled electrical stimulation on biological cells considering model uncertainties. *Scientific Reports* 12, 4744. doi:10.1038/s41598-022-08279-w
